# A switch in the mode of Wnt signaling orchestrates the formation of germline stem cell differentiation niche in *Drosophila*

**DOI:** 10.1371/journal.pgen.1007154

**Published:** 2018-01-25

**Authors:** Maitreyi Upadhyay, Michael Kuna, Sara Tudor, Yesenia Martino Cortez, Prashanth Rangan

**Affiliations:** 1 Department of Biological Sciences/RNA Institute, University at Albany SUNY, Albany, New York, United States of America; 2 Albany Medical College, Albany, New York, United States of America; 3 Tufts University School of Medicine, Boston, Massachusetts, United States of America; University of Bristol, UNITED KINGDOM

## Abstract

Germline stem cell (GSC) self-renewal and differentiation into gametes is regulated by both intrinsic factors in the germ line as well as extrinsic factors from the surrounding somatic niche. dWnt4, in the escort cells of the adult somatic niche promotes GSC differentiation using the canonical β-catenin-dependent transcriptional pathway to regulate escort cell survival, adhesion to the germ line and downregulation of self-renewal signaling. Here, we show that in addition to the β-catenin-dependent canonical pathway, dWnt4 also uses downstream components of the Wnt non-canonical pathway to promote escort cell function earlier in development. We find that the downstream non-canonical components, RhoA, Rac1 and cdc42, are expressed at high levels and are active in escort cell precursors of the female larval gonad compared to the adult somatic niche. Consistent with this expression pattern, we find that the non-canonical pathway components function in the larval stages but not in adults to regulate GSC differentiation. In the larval gonad, dWnt4, RhoA, Rac1 and cdc42 are required to promote intermingling of escort cell precursors, a function that then promotes proper escort cell function in the adults. We find that dWnt4 acts by modulating the activity of RhoA, Rac1 and cdc42, but not their protein levels. Together, our results indicate that at different points of development, dWnt4 switches from using the non-canonical pathway components to using a β-catenin-dependent canonical pathway in the escort cells to facilitate the proper differentiation of GSCs.

## Author summary

Germ line association with the somatic cells is critical for various aspects of germ cell biology, including migration, self-renewal and differentiation. In *Drosophila* females, soma–germ line association begins during embryogenesis and continues until the mature egg is formed. In the adult, the somatic escort cells promote differentiation of the germline stem cell daughter using Wnt signaling. dWnt4, a Wnt ligand, acts in an autocrine manner in these escort cells, using the canonical pathway to regulate survival, division and encapsulation of the stem cell daughter, a function critical for differentiation. Here, we show at an earlier stage, in the larvae, the same ligand uses components of Wnt non-canonical pathway, RhoA, Rac1 and cdc42, to regulate proper mingling of escort cell precursors between the germ cells. Thus, dWnt4 uses different modules of signaling at different points in development to promote cell movement and control cytoplasmic protrusions. As Wnts have been associated with cancers, understanding how Wnts modulate cell movement by switching on and off different modules may lead to insights into the etiology and progression of cancers.

## Introduction

Stem cell self-renewal and differentiation are critical for maintaining the organ systems of multicellular organisms. Loss of stem cell self-renewal leads to aging, due to an inability to replenish these organs; while loss of differentiation leads to tumors, which can progress toward diseases such as cancer [1–3]. Thus, identifying triggers of stem cell differentiation is pivotal for understanding the etiology of degenerative diseases and cancer. Germline stem cells (GSCs) self-renew and differentiate to produce gametes [4–7]. The balance between self-renewal and differentiation is critical for a steady supply of gametes for increased reproductive success. Dysregulation of GSC self-renewal and differentiation manifests itself as changes in fecundity without markedly altering the organism’s growth or survival. Additionally, processes regulating GSC differentiation are conserved in other stem cell systems [8–10]. Therefore, GSCs make an excellent system to identify triggers of stem cell differentiation.

**Fig 1:**
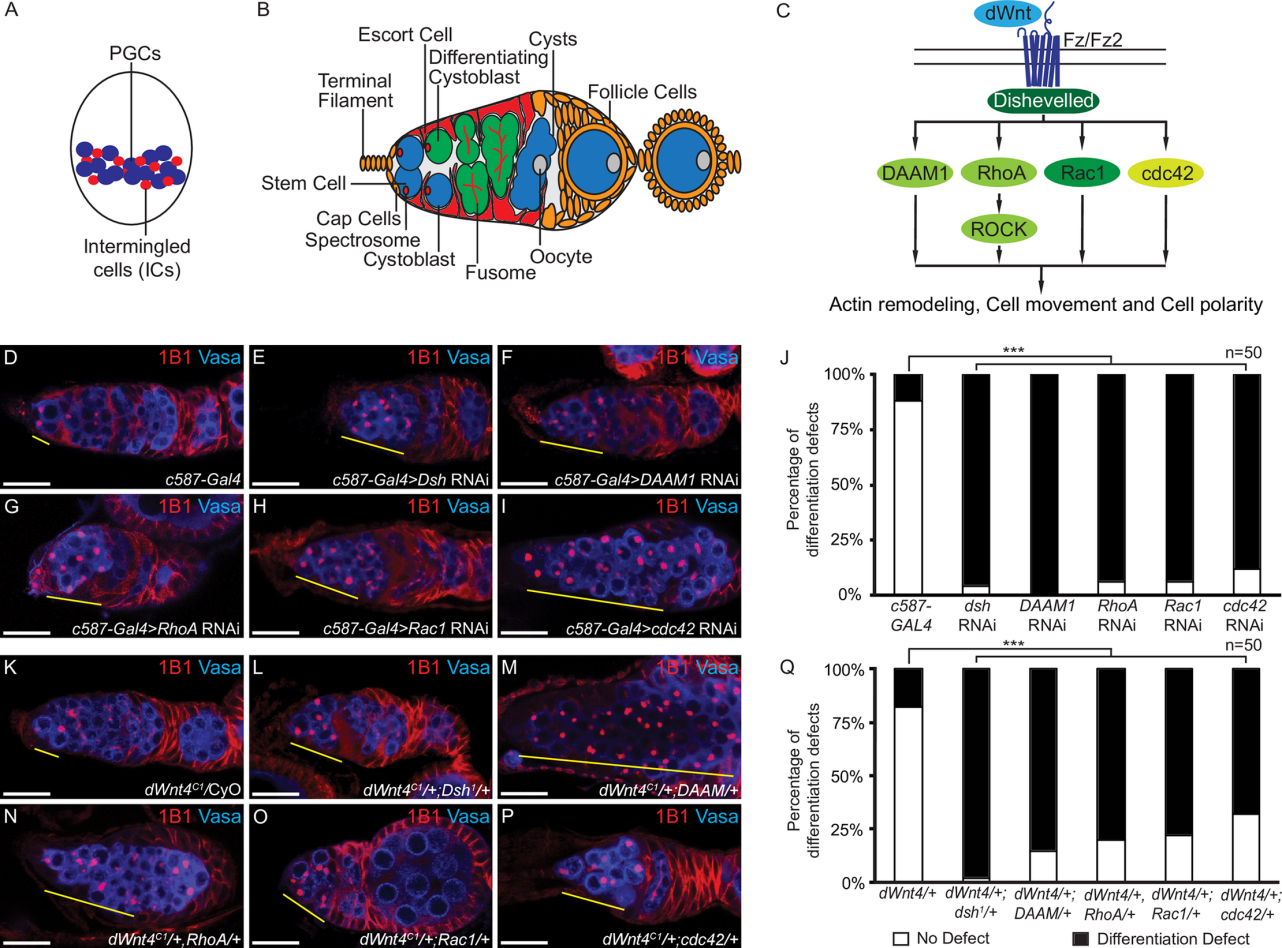
The downstream Wnt non-canonical components are required in the escort cells for proper germline stem cell differentiation. (A) Schematic of a female late larval gonad. The primordial germ cells (PGCs) (blue) are interspersed with the intermingled cells (ICs) (red), precursors of escort cells. (B) A schematic of the *Drosophila* female germarium present at the anterior end of the ovarioles. The germ line consists of germline stem cells (GSCs) (blue) that are attached to the self-renewal somatic niche made by the terminal filament and cap cells (orange). The GSCs divide to give rise to the cystoblast (blue) that differentiates on expression of a differentiating factor, Bam. The differentiating cystoblast (green) undergoes four incomplete mitotic divisions (green) to give rise to a sixteen-cell cyst (green), one of which becomes an oocyte (grey). The differentiating somatic niche made by the escort cells (red) encapsulates the cystoblast and the differentiating progeny. C) An illustration of the dWnt non-canonical pathway. dWnt binds to its receptor, Frizzled/Frizzled2 (Fz/Fz2) and activates DAAM1, RhoA, Rac1, and cdc42, downstream of Dsh. RhoA activates ROCK. These pathways are required for actin remodeling, cell movement and cell polarity. (D-I) Germaria of *c587-GAL4* (control) and *dsh*, *DAAM1*, *RhoA*, *Rac1* and *cdc42* depleted escort cells stained with 1B1 (red) and Vasa (blue) showing an accumulation of >3 undifferentiated cells in *dsh*, *DAAM1*, *RhoA*, *Rac1* and *cdc42* mutants (yellow line). (J) Percentage of the germaria with >3 spectrosomes in *c587-GAL4*, *dsh*, *DAAM1*, *RhoA*, *Rac1* and *cdc42* depleted escort cells showing a significant difference in mutants (n = 50). (K-P) *dWnt4* heterozygote; *dWnt4*/*dsh*^*1*^; *dWnt4*/*DAAM*; *dWnt4*,*RhoA*; *dWnt4*/*Rac1* and *dWnt4*/*cdc42*^*3*^ trans-heterozygote stained with 1B1 (red) and Vasa (blue) showing an accumulation of >3 undifferentiated cells (yellow line) in the trans-heterozygotes. (Q) Percentage of the differentiation defects in *dWnt4* heterozygote, *dWnt4*/*dsh*^*1*^; *dWnt4*/*DAAM*; *dWnt4*,*RhoA*; *dWnt4*/*Rac1* and *dWnt4*/*cdc42*^*3*^ trans-heterozygote showing a significant difference in trans-heterozygotes (n = 50). Scale bar for all images is 20μm.

*Drosophila* GSCs are well characterized and genetically tractable [5]. GSC development starts during the larval stages. The female larval gonad is made up of the somatic niche and primordial germ cells (PGCs) [11,12]. The late larval somatic niche is comprised of intermingled cells (ICs), the terminal filament and the cap cells [11–14]. The PGCs that give rise to the GSCs in the adults are interspersed with ICs, precursors of the adult escort cell [11,15,16] **(Fig 1A)**. Most of the PGCs remain undifferentiated during the larval stage [15,17,18]. As development progresses, the larval gonad transforms first into a pupal gonad and then into an adult ovary. During this transition, the PGCs closest to the cap cells acquire a stem cell fate and become GSCs while the rest directly differentiate [15,18]. Each adult ovary consists of 16–18 individual units called ovarioles. GSCs are located in the germarium, present at the anterior end of each ovariole [19]. GSCs divide to give rise to a self-renewed GSC and a stem cell daughter, the cystoblast (CB) **(Fig 1B)**. The CB expresses a differentiating factor called Bag of marbles (Bam), undergoes differentiation and four incomplete divisions to form a sixteen-cell cyst. Of the sixteen cells, fifteen become nurse cells and one is specified as an oocyte [20–24] **(Fig 1B)**. Loss of GSC differentiation results in loss of or delayed progression towards becoming an oocyte.

Soma-germ line interaction is critical for proper GSC self-renewal and differentiation [8,25,26]. Both PGCs in the larval gonad and GSCs in the adults are surrounded by somatic cells that constitute the somatic niche. Close contact and coordinated signaling between the surrounding somatic niche and the germ line is pivotal for self-renewal and differentiation [26,27]. In the larval stages, ICs regulate PGC proliferation [11]. Additionally, proper intermingling of ICs in the larval gonad promotes proper escort cell function in the adults [28,29]. In the adults, the somatic niche can be broadly divided into two regions; the self-renewal niche and the differentiation niche [26,30]. The terminal filament and cap cells comprise the self-renewal niche that is required for regulating GSC self-renewal **(Fig 1B)** [24,26,31–36]. Loss of adherens junction proteins, such as *DE-Cadherin* and *Armadillo/β-catenin*, in either germline or somatic cells within the self-renewal niche, leads to loss of contact and thus loss of GSC self-renewal [37,38]. The differentiation niche, formed by the escort cells, makes extensive contact with CBs by means of escort cell protrusions that encapsulate the CB and promote GSC differentiation **(Fig 1B)** [39–41]. Thus, soma-germline contact throughout development is a critical extrinsic cue that controls GSC differentiation.

Signaling from the somatic niche coordinates the balance of self-renewal and differentiation. In the larval gonad, EGF/Spitz signaling from the PGCs is required for IC survival and intermingling [11]. In the adult germaria, *decapentaplegic (dpp)* signaling in the self-renewal niche regulates GSC self-renewal by phosphorylating the transcriptional regulator, mothers against dpp (pMAD), to repress expression of the differentiation factor, Bam [20,24,31]. Ecdysone and dWnt4, among others, have been shown to function in the differentiation niche to promote the differentiation of the CB. Ecdysone signaling in the escort cells regulates the formation of escort cell protrusions through an unknown mechanism [42,43]. dWnt4 autocrine signaling in the escort cells is required for escort cell survival, repression of *dpp* expression and for formation of escort cell protrusions to encapsulate the CB [44–46].

Canonical Wnt signaling plays a critical role in the adult differentiation niche. Wnts can signal through either a canonical or a non-canonical pathway to affect downstream changes [47]. In the escort cells, dWnt4 is known to regulate differentiation through the canonical pathway [44–46]. dWnt4 binds to the receptor, Frizzled2 and co-receptor, Arrow (Arr), and relays a signal to the multidomain protein, Dishevelled (Dsh), leading to stabilization of β-catenin [47–50]. β-catenin, a cytoskeletal protein that is also a transcription factor, translocates into the nucleus and initiates transcription of downstream targets including Frizzled3 (Fz3) [51–57]. Depletion of any of the canonical pathway factors, such as *β-catenin* or co-receptor *arr*, specifically in the escort cells leads to loss of CB differentiation by regulating escort cell number [44–46]. Additionally, loss of *dWnt4* leads to downregulation of a Wnt canonical reporter, Fz3 promoter fused to RFP (Fz3RFP), and adhesion molecules, β-catenin, Innexin2 and DE-Cadherin [46]. These adhesion molecules promote escort cell encapsulation of the CB that is required for its differentiation [46]. Thus, dWnt4 uses components of the canonical pathway to control both the number and function of escort cells.

Wnt ligands, such as dWnt4, can modulate the Planar Cell Polarity (PCP) of cells [58]. Polarity within a plane of individual cells or of tissues is known as PCP. PCP can specify the proximal and distal end of a cell, as in the *Drosophila* wing, or the dorsal-ventral and anterior-posterior side of a cell, as in the *Drosophila* eye [58–64]. Two systems are known to independently regulate PCP–the core components and the Daschous (Ds)/Fat (Ft) system [65,66]. The core components consist of six proteins—Frizzled (Fz), Dishevelled (Dsh), Diego (Dgo), Van Gogh (Vang)/Strabismus (Stbm), Prickle (Pk), and Flamingo (Fmi). These proteins form two complexes that are asymmetrically arranged on the opposite ends of a cell–the Fz, Dsh and Dgo, and the Vang/Stbm, Pk complex. Fmi is enriched on both sides of the cell and stabilizes the complexes [59–61,67]. Small Rho GTPases such as Ras homolog gene family, member A (RhoA), Ras-related C3 botulinum toxin substrate 1 (Rac1) and Cell division control protein 42 (cdc42) can function as the downstream effectors of the core complex [60,68–70]. The Ds/Ft system consists of two atypical transmembrane cadherin proteins–Ds and Ft. Ds and Ft are present on opposite ends of each cell and bind to each other at cell-cell junctions. A golgi associated kinase, Four Jointed (Fj) phosphorylates the extracellular domains of Ds and Ft and thereby promotes their binding [66,71]. The role of the core components and the Ds/Ft system has been well established in specifying PCP in the wing and the eye.

Binding of Wnt to Frizzled2, can also activate pathways downstream of Dsh: Dishevelled Associated Activator of Morphogenesis 1 (DAAM1), Ras homolog gene family member A (RhoA), Rac1 and cdc42 pathway. This signaling cascade is also referred to as Wnt/Fz non-canonical pathway. Activated RhoA activates Rho associated kinase (ROCK/dRok) [47,61,72–75]. Together, these pathways regulate the cell division, cytoskeleton, cell movement and cell polarity [76–81] **(Fig 1C)**. It has been previously shown that, like *dWnt4* mutants, flies carrying mutations in *dsh*^*1*^, a PCP specific allele, show disrupted oogenesis [82]. In contrast, loss of other genes that regulate PCP such as *fz*, *vang/stbm*, *fmi*, *dgo*, and *pk* do not show such a defect, suggesting that PCP might not be operating during oogenesis [82]. However, it is not known if and how downstream components of the Wnt/Fz non-canonical pathway promote oogenesis.

Here, we find that *RhoA*, *Rac* genes and *cdc42*, the downstream Wnt non-canonical components, but not members of the core components that define PCP, such as *vang*, *ds*, *fz* and *ft*, regulate CB differentiation. We find that *dWnt4* genetically interacts with the downstream non-canonical components: *dsh*^*1*^, *DAAM1*, *RhoA*, *Rac1* and *cdc42*. RhoA, Rac1 and cdc42 are expressed at higher levels and are more active in the ICs of the late larval gonad than in the adult escort cells. Conversely, the Wnt canonical reporter is not expressed in the late larval stages but is expressed in the adults. Consistent with this observation, we find that these downstream non-canonical pathway components are required in the late larval stages for regulating intermingling of ICs with the germ cells but their function is not essential in the adults to regulate differentiation. Additionally, we find that *dWnt4* regulates the activity, but not the protein levels, of RhoA, Rac1 and cdc42 in the larval gonad. Thus, dWnt4 regulates assembly of the somatic differentiation niche of the GSCs by switching the mode of signaling during development.

## Results

### *dWnt4* acts through the downstream non-canonical pathway components to regulate differentiation

To determine if the Wnt non-canonical pathway components regulate differentiation, we depleted these components in the escort cells. We made use of RNA interference (RNAi) in conjunction with *c587-GAL4*, which is expressed in the escort cells, to deplete *dsh*, *DAAM1*, *RhoA*, *Rac1* and *cdc42* [43,83,84]. Both control and mutant germaria were stained with 1B1 and Vasa. 1B1 marks the endoplasmic reticulum rich organelle, the spectrosome, in undifferentiated GSCs and CBs as well as in differentiating CBs; and it marks the branched structures, the fusomes, in differentiated cysts [85]. 1B1 also marks somatic cell membranes [86]. Vasa, an RNA helicase, marks the germ line [87]. We assayed for differentiation defects, defined as an accumulation of greater than 3 undifferentiated cells marked by spectrosomes. Depletion of *dsh*, *DAAM1*, *RhoA*, *Rac1* and *cdc42* resulted in germline differentiation defects compared to control **(Fig 1D–1J) (S1A Fig) (Table 1)**. We also depleted the mediators that define the PCP of a cell utilizing previously validated RNAi lines in conjunction with *c587-GAL4*. We found that for *vang*, *ds*, *ft* and *fz*, we did not observe any significant differentiation defect [42,88–90] **(S1B–S1I Fig) (Table 1)**. These results are consistent with Cohen *et al*’s observation that mutations in genes that regulate PCP of a cell, such as *fz*, *stbm*, *fmi*, *dgo* and *pk*, do not lead to defects in oogenesis [82]. Thus, we can conclude that downstream components of the Wnt non-canonical pathway such as *dsh*, *DAAM1*, *RhoA*, *Rac1* and *cdc42* are required in the escort cells for promoting GSC differentiation. Additionally, these results also suggest that the core components that mediate PCP such as *vang/stbm*, *ds*, *fz* and *ft* do not play a significant role in the escort cells to regulate GSC differentiation.

**Table 1:** Quantitation of undifferentiated cells in mutants compared to control germaria.

Genotype	Spectrosomes	n	P-value / %Differentiation defect
*c587-GAL4*	2.9 ± 0.7	50	
*c587-GAL4>dishevelled RNAi*	7.0 ± 1.4	50	2.69663E-29
*c587-GAL4>dDAAM1* RNAi	7.8 ± 1.5	50	4.7793E-38
*c587-GAL4>RhoA* RNAi	7.2 ± 2.5	50	4.6713E-24
*c587-GAL4>Rac1* RNAi	4.0 ± 1.5	50	4.38037E-11
*c587-GAL4>cdc42* RNAi	7.2 ± 4.5	50	6.05032E-12
*c587-GAL4>van gogh* RNAi	2.6 ± 1	50	0.194156
*c587-GAL4>daschous* RNAi	3.0 ± 1.3	50	0.333138
*c587-GAL4>frizzled* RNAi	2.5 ± 1	50	0.0907
*c587-GAL4>fat* RNAi	2.9 ± 0.8	50	0.353701
*dWnt4*/CyO	2.7 ± 1.1	50	17.5% Differentiation Defect
*dsh*^*1*^*/+*	2.8± 1.3	50	
*DAAM**/*Fm7	2.4 ± 1	50	16% Differentiation Defect
*RhoA**/*CyO	2.9 ± 1.2	50	24% Differentiation Defect
*Rac1/*TM6	2.7 ± 1.1	50	22% Differentiation Defect
*Cdc42*^*3*^*/*Fm6	3.9 ± 1.7	50	28% Differentiation Defect
*DAAM*/+;*dWnt4/+*		50	Z-score = 7.1376, P-value = 0 (compared to *dWnt4*/CyO) Z-score = 8.0064, P-value = 0 (compared to *DAAM*/Fm7)
*dsh*^*1*^/+;*dWnt4/+*		50	Z-score = 7.1376, P-value = 0 (compared to *dWnt4*/CyO) 1.47184E-13(compared to *dsh*^*1*^*/+)*
*RhoA*/+,*dWnt4/+*		50	Z-score = 4.0718, P-value = 0 (compared to *dWnt4*/CyO) Z-score = 3.4338, P-value = 0.0006 (compared to *RhoA*/CyO)
*Rac1/+;dWnt4/+*		50	Z-score = 4.4236, P-value = 0 (compared to *dWnt4*/CyO) Z-score = 4.2418, P-value = 0 (compared to *Rac1*/TM6)
*dsh*^*1*^	4.1 ± 1.5	50	2.30222E-05 (compared to *dsh*^*1*^/+)
*dsh*^*1*^*/+; **RhoA**/+*	4.1 ± 1.3	50	5.9375E-06(compared to *dsh*^*1*^/+) 2.7340E-06 (compared to *RhoA*/CyO)
*dsh*^*1*^*/+; Rac1/+*	4.1 ± 1.5	50	7.4036E-05 (compared to *dsh*^*1*^/+) 1.83104E-05 (compared to *Rac1*/TM6)
*Cdc42*^*3*^/+;*dWnt4/+*		50	Z-score = 4.7801, P-value = 0 (compared to *dWnt4*/CyO) Z-score = 3.0298, P-value = 0.00032 (compared to *Cdc42*^*3*^*/*Fm6)

DAAM1, RhoA, Rac1 and cdc42 are critical proteins required for various basic cellular processes, such as progression of the cell cycle and cell movement [76–80,91]. It is not surprising that they are required in the escort cells for proper function. We asked if these proteins act downstream of dWnt4 to promote differentiation. To answer this, we generated trans-heterozygous flies that contained one genetically reduced copy of *dWnt4* and one reduced copy of *dsh*, *DAAM*, *RhoA*, *Rac1* or *cdc42* [92–96]. Dsh is a multi-domain protein that uses distinct domains to interact with either the Wnt canonical or the Wnt non-canonical proteins [97]. We used *dsh*^*1*^, a PCP specific allele, to test if dWnt4 uses the downstream components of the PCP system [68,98]. If the downstream PCP components act through the same pathway, then these trans-heterozygotes could exhibit GSC differentiation defects, but if they act parallel or independent of dWnt4, then these trans-heterozygotes should not exhibit differentiation defects. We stained these trans-heterozygote ovaries for 1B1 and Vasa to assay for differentiation defects. Compared to 17.5% (n = 50) of heterozygous *dWnt4* germaria, 98% (n = 50) of *dsh*^*1*^*;dWnt4* trans-heterozygote, 85% (n = 50) of *DAAM;dWnt4* trans-heterozygote, 80% (n = 50) of *RhoA*,*dWnt4* trans-heterozygote, 78% (n = 50) of *Rac1;dWnt4* trans-heterozygote, and 68% (n = 50) of *cdc42*^*3*^*;dWnt4* trans-heterozygote germaria showed accumulation of spectrosomes **(Fig 1K–1Q) (S2A–S2E Fig) (Table 1)**. As Dsh acts downstream of Wnt signaling, we also stained *dsh*^*1*^, *RhoA* and *Rac1* heterozygous, and trans-heterozygous flies with one mutant copy of *dsh*^*1*^ and one mutant copy of *RhoA* or *Rac1* for 1B1 and Vasa. Compared to heterozygous *dsh*^*1*^, *RhoA* and *Rac1* germaria, we found significantly higher number of spectrosomes in *dsh*^*1*^ mutants, *dsh*^*1*^*;RhoA* trans-heterozygotes, and *dsh*^*1*^*;Rac1* trans-heterozygotes **(S2D–S2H Fig) (Table 1)**. Taken together, these results suggest that *dWnt4* can function via *dsh* and the downstream Wnt non-canonical components to regulate GSC differentiation.

### Downstream Wnt non-canonical components regulate CB encapsulation to promote differentiation

If dWnt4 acts through the downstream Wnt non-canonical components to regulate GSC differentiation, then *dWnt4* mutants and *RhoA*, *Rac1* and *cdc42* depleted escort cell mutants should phenocopy each other. Loss of *dWnt4* leads to an accumulation of pre-CBs that do not express Bam due to loss of escort cell encapsulation and a reduction of escort cell number [44–46]. To test if depletion of the downstream non-canonical components in the escort cells also results in an accumulation of pre-CBs, we stained control and mutants for pMAD, which marks the GSCs, and used GFP under the control of *bam* promoter to analyze bam transcription and hence mark the differentiating progeny [99]. In addition, we also stained control and mutants for pMAD and BamC that marks proper translation of *bam* in the differentiating progeny. Similar to *dWnt4* mutants, *RhoA*, *Rac1* and *cdc42* depleted escort cell mutants showed an accumulation of pMAD negative, and BamGFP and BamC negative cells, suggesting that these mutants accumulate pre-CBs **(S3A–S3E Fig) (S4A–S4D2 Fig)**. Interestingly, in addition to early pre-CBs, *cdc42* mutants also showed an accumulation of BamGFP and BamC positive CBs, suggesting that *cdc42* may have an additional role in promoting CB differentiation.

Pre-CBs that accumulate in *dWnt4* mutants are capable of differentiation upon ectopic expression of *bam*. To test if the undifferentiated germ cells that accumulate due to depletion of downstream components of PCP in the escort cells are also capable of differentiating, we ectopically expressed *bam* by using a transgene that expresses *bam* under the control of heat-shock promoter (*hs-bam*) [21]. We found, post heat-shock, these mutants showed loss of undifferentiated cells and an accumulation of cysts, as marked by the presence of fusomes comparable to the control (90% for *c587-GAL4*; 86% for *c587-GAL4>RhoA* RNAi, P-value = 0.5353; 76% for *c587-GAL4>Rac1* RNAi, P-value = 0.0629 and 92% for *c587-GAL4>cdc42* RNAi, P-value = 0.7263 {for all n = 50}) **(S5A–S5E Fig)**. We also analyzed the expression of Bruno in *RhoA*, *Rac1* and *cdc42* depleted escort cell mutants that carry the *hs-bam* transgene, without heat-shock and post heat-shock. Bruno, a translational repressor, is expressed at very low levels in the undifferentiated cells but is expressed at high levels post-differentiation from 16-cell cysts onwards [100,101]. We found, post heat-shock, cysts in *Rac1* and *cdc42* mutants that carry the *hs-bam* transgene expressed Bruno, while cysts in *RhoA* mutants carrying the *hs-bam* transgene only weakly expressed Bruno **(S5F–S5M1 Fig)**. Altogether, these results suggest that the downstream Wnt non-canonical pathway components are required extrinsically in escort cells, to promote differentiation in the germ line.

**Fig 2:**
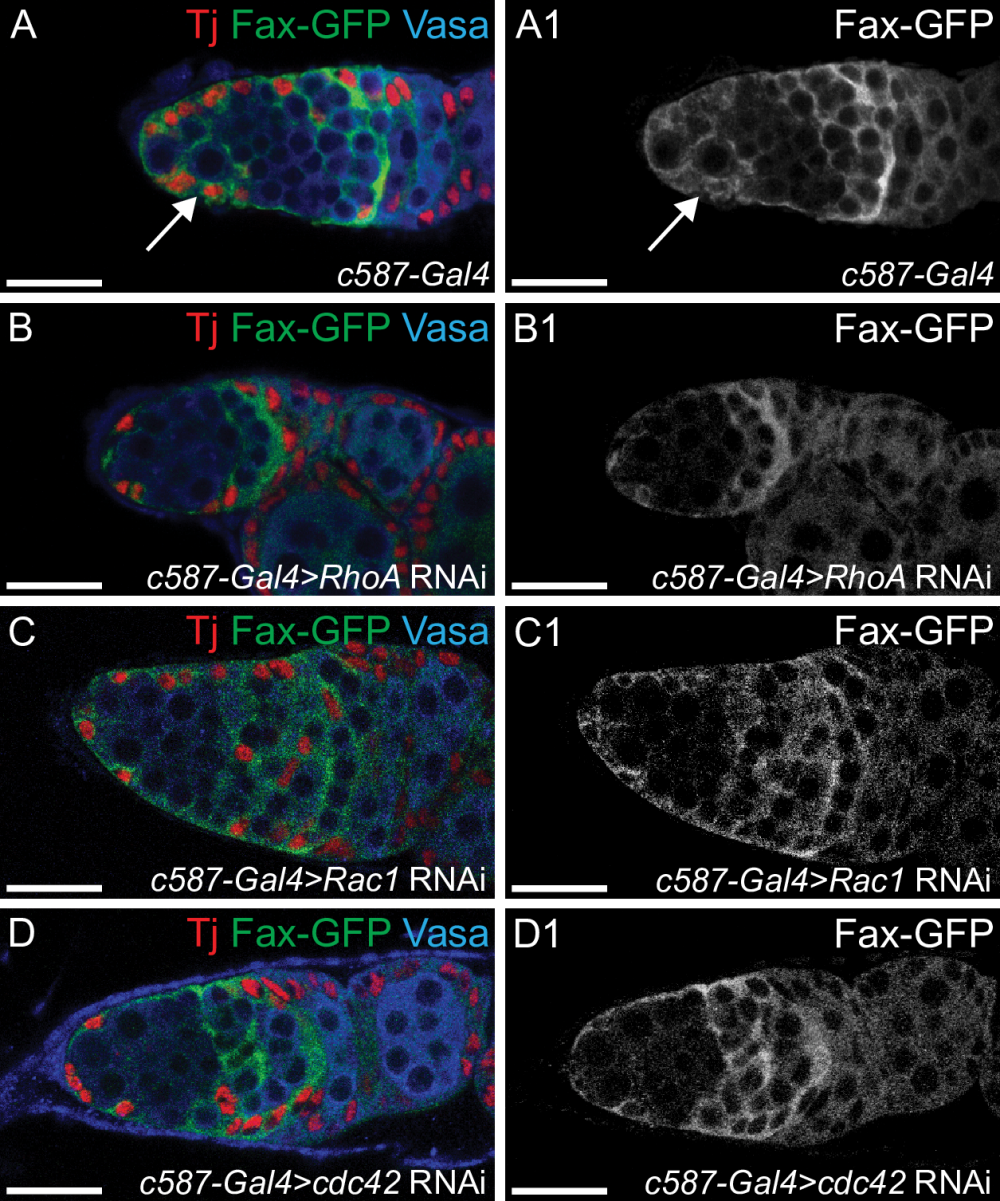
The downstream Wnt non-canonical pathway components are required in the escort cells for cystoblast encapsulation. (A-D1) Germaria of *c587-GAL4*, *RhoA*, *Rac1* and *cdc42* depleted escort cells stained with Tj (red), GFP (green) and Vasa (blue) showing loss of encapsulation in *RhoA*, *Rac1* and *cdc42* depleted escort cells. Fax-GFP marks somatic cell membranes. GFP channel is shown in A1, B1, C1 and D1. Scale bar for all images is 20μm.

Loss of escort cell number and their encapsulation results in loss of pre-CB differentiation [39–41,102]. To determine if loss of the downstream Wnt non-canonical pathway components leads to loss of encapsulation, we visualized the cytoplasmic protrusions using FaxGFP, a somatic cell membrane marker [39]. It has been previously demonstrated that overexpression in the escort cells of dominant-negative Rho (*Rho*^*DN*^), that disrupts Rho function, leads to loss of escort cell encapsulation [40]. We found that, similar to *dWnt4* mutants and germaria where *Rho*^*DN*^ was overexpressed in the escort cells, depletion of *RhoA*, *Rac1* and *cdc42* in the escort cells also resulted in loss of encapsulation **(Fig 2A–2D1)**. To determine if this was due to decreased number of escort cells, we counted the number of Tj positive escort cells in these mutants. We found that these mutants exhibited significantly lower number of escort cells (16.5 ± 2.1 for *c587-GAL4*; 9.9 ± 3.7 for *c587-GAL4>RhoA* RNAi, P-value = 0.00013; 11 ± 1.5 for *c587-GAL4>Rac1* RNAi, P-value = 4.1403E-06 and 9.3 ± 2.2 for *c587-GAL4>cdc42* RNAi, P-value = 9.5478E-07 {for all n = 10}). However, the escort cells that were present in the germaria failed to extend cytoplasmic protrusions. These results demonstrate that like *dWnt4*, downstream Wnt non-canonical pathway components in the escort cells regulate both encapsulation and their numbers, and thus CB differentiation [44–46]. These results taken together suggest that dWn4 acts through Dsh and the downstream Wnt non-canonical pathway components to regulate differentiation.

### Components of Wnt non-canonical pathway are expressed at high levels and are active in the larval gonad

Wnt proteins are known to regulate signaling through either the canonical or the non-canonical pathway [47]. Surprisingly, our data suggests that dWnt4 also acts through the non-canonical pathway components in the escort cells. There is little precedent for canonical and non-canonical Wnt pathways acting in the same cell at the same time. We therefore hypothesized that either dWnt4 signals via the canonical or non-canonical arm in distinct subsets of escort cells or at different stages of escort cell development. If there are subsets of cells that respond to canonical signaling but not to non-canonical signaling, then we could observe the canonical reporter to be present only in some escort cells. If the regulation is temporal, we hypothesized that we could see differences in either levels or activity of canonical and non-canonical reporters as a function of development.

**Fig 3:**
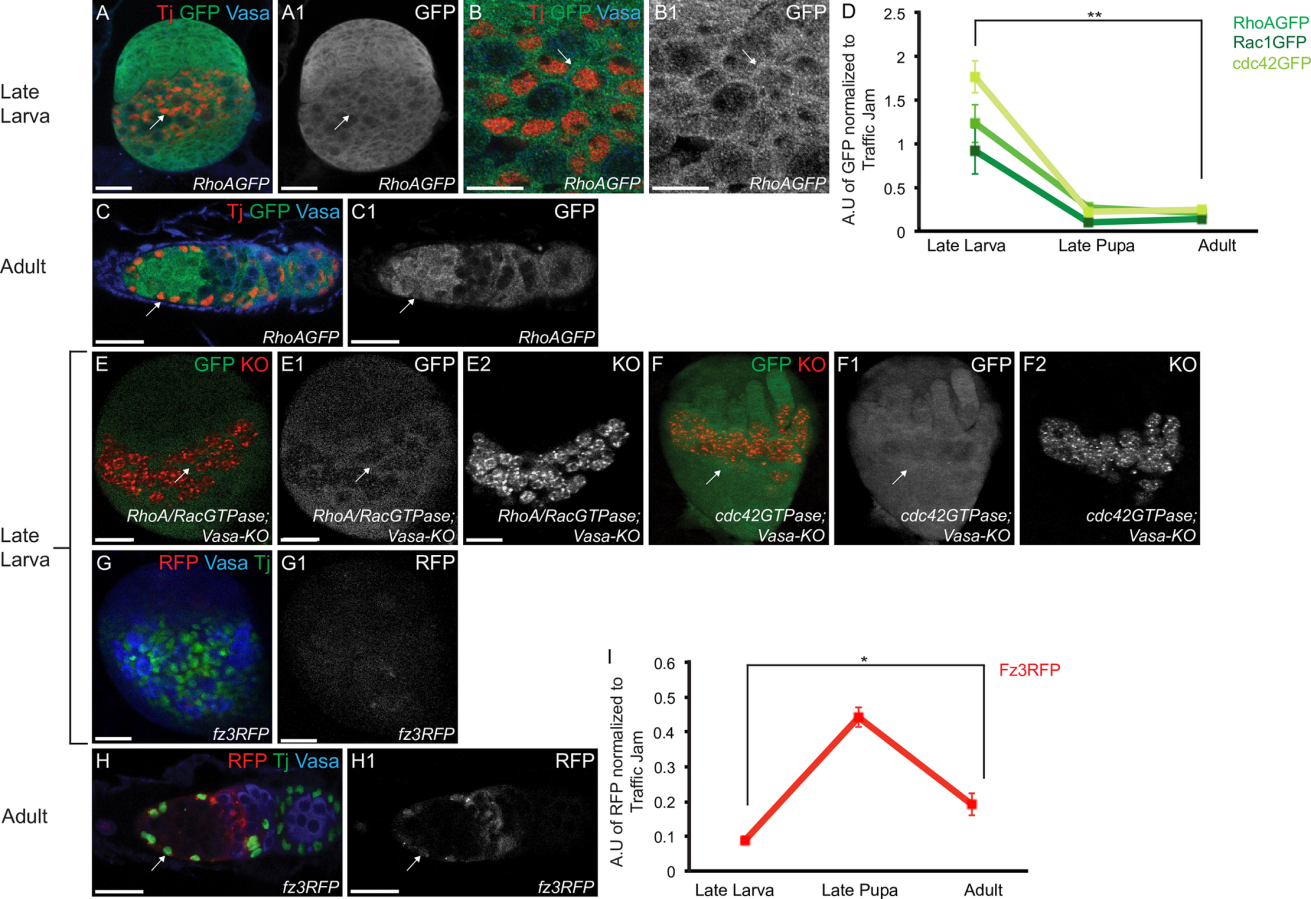
The downstream Wnt non-canonical pathway components are temporally regulated. (A-B1) Larval gonad of transgenic fly with GFP tagged to *RhoA* stained for Tj (red), Vasa (blue) and GFP (green) showing high expression of RhoA in the ICs. 63x of larval gonad is shown in B and B1. GFP channel is shown in A1 and B1. (C-C1) Adult germarium of transgenic fly with GFP tagged to *RhoA* stained for Tj (red), Vasa (blue) and GFP (green) showing low expression of RhoA in the adult escort cells. GFP channel is shown in C1. (D) Quantification (n = 7) of GFP in the ICs, pupal escort cells and adult escort cells showing that the downstream Wnt non-canonical components are highly expressed in the ICs in comparison to the adult escort cells. (E-F2) Larval gonads of transgenic flies that report active form of RhoA/Rac (E-E2) and cdc42 (F-F2). While RhoA/Rac is active in the ICs, cdc42 is highly active in the ICs and terminal filament and comparatively less active in the Primordial Germ Cells (PGCs). Germline is marked by Vasa-KO. GFP channels and KO channels are shown in E1, E2, F1 and F2. (G-G1) Larval gonad stained for 1B1 (green), Vasa (blue) and RFP (red) showing low expression of the Wnt canonical reporter, Frizzled3 in the ICs. RFP channel is shown in G1. (H-H1) Adult germarium stained for Tj (green), Vasa (blue) and RFP (red) showing high expression of Frizzled3 in the adult escort cells. RFP channel is shown in H1. (I) Quantification (n = 7) of RFP in the ICs, pupal escort cells and adult escort cells showing that the canonical reporter is expressed at higher levels in the adult escort cells in comparison to the ICs. Scale bar for B and B1 is 10μm. Scale bar for all other images is 20μm.

To test these hypotheses, we analyzed the expression patterns of downstream Wnt non-canonical pathway components and a Wnt canonical reporter during late larval stages, late pupal gonads and in the adult ovaries. Transgenic lines with GFP tagged *RhoA*, *Rac1* or *cdc42* that report endogenous expression were used for the Wnt non-canonical components [103]. These reporters were stained for their respective fluorescent proteins and Tj, which marks ICs in the larval gonad and all somatic cells, except for the terminal filament, in the adult gonad [29]. Vasa was used to mark the germ line. We found that RhoA, Rac1 and cdc42 showed high expression in the larval gonad **(Fig 3A–3B1 and 3D) (S6A–S6D1 Fig)**. In contrast, we found that RhoA, Rac1 and cdc42 were expressed at low levels in the escort cells of the pupal and adult germaria **(Fig 3C and 3D) (S6E–S6I1 Fig)**. This different expression pattern suggests that the Wnt non-canonical pathway members are regulated in a temporal manner in the escort cells and their precursors during oogenesis.

RhoA, Rac1 and cdc42 are members of the Rho family of small GTPases. These proteins are inactive when bound to GDP and active when bound to GTP [104,105]. The active forms of these proteins regulate various downstream signaling cascades [76,77,106,107]. We observed that downstream components of the Wnt non-canonical pathway, RhoA, Rac1 and cdc42 are expressed at high levels in the larval gonad. We asked if these proteins are present in their activated form in the larval gonad. To test this, we performed live-imaging of larval gonads of transgenic lines that report the active form of RhoA/Rac and cdc42 [108]. In these biosensors, GFP is fused to the Rho family GTPase Binding Domain (RBD) of downstream effector proteins. Expression of GFP suggests the presence of active forms of these proteins. For RhoA/Rac activity, the downstream effector protein, Protein Kinase N (Pkn) was fused to GFP and placed under *squash* (*sqh*) promoter [108]. For cdc42 activity, the cdc42 binding domain of Wiskott-Aldrich Syndrome protein (WASp) was fused with GFP and this was placed under the control of *sqh* promoter [108]. In order to observe the germ cells in these biosensors, we used kusabira-orange (KO) fused germline marker, Vasa-KO [109,110]. Similar to the expression pattern of the GFP tagged RhoA, Rac1 and cdc42 we observed the presence of active form of RhoA, Rac1 and cdc42 in the ICs of the larval gonad **(Fig 3E–3F2)**. We also asked if these proteins are present in their active forms in the escort cells of the adult germaria. We found that RhoA/Rac activity was attenuated and cdc42 was present in its activated state in the escort cells, albeit at lower levels compared to the larval gonad **(S7A–S7C Fig)**. Together, these results demonstrate that the Wnt non-canonical components are not only present, but also active in the ICs. To determine the activity of the canonical pathway in the larval gonad, we used Fz3RFP [57]. We found that while Fz3RFP showed no expression or was expressed at background levels in the ICs of the larval gonad, it was expressed at higher levels in the escort cells of pupal and adult stages [46,111,112] **(Fig 3G–3I) (S7D–S7D1 Fig)**. Additionally, all the escort cells in the adult germarium expressed the canonical reporter (n = 10, 99.5% Tj positive cells expressed Fz3RFP). Thus, we concluded that Wnt canonical and Wnt non-canonical pathway components, and their activity, are temporally regulated in escort cells and their precursors during oogenesis.

### Components of the Wnt non-canonical pathway play a critical role in the larval gonad

**Fig 4:**
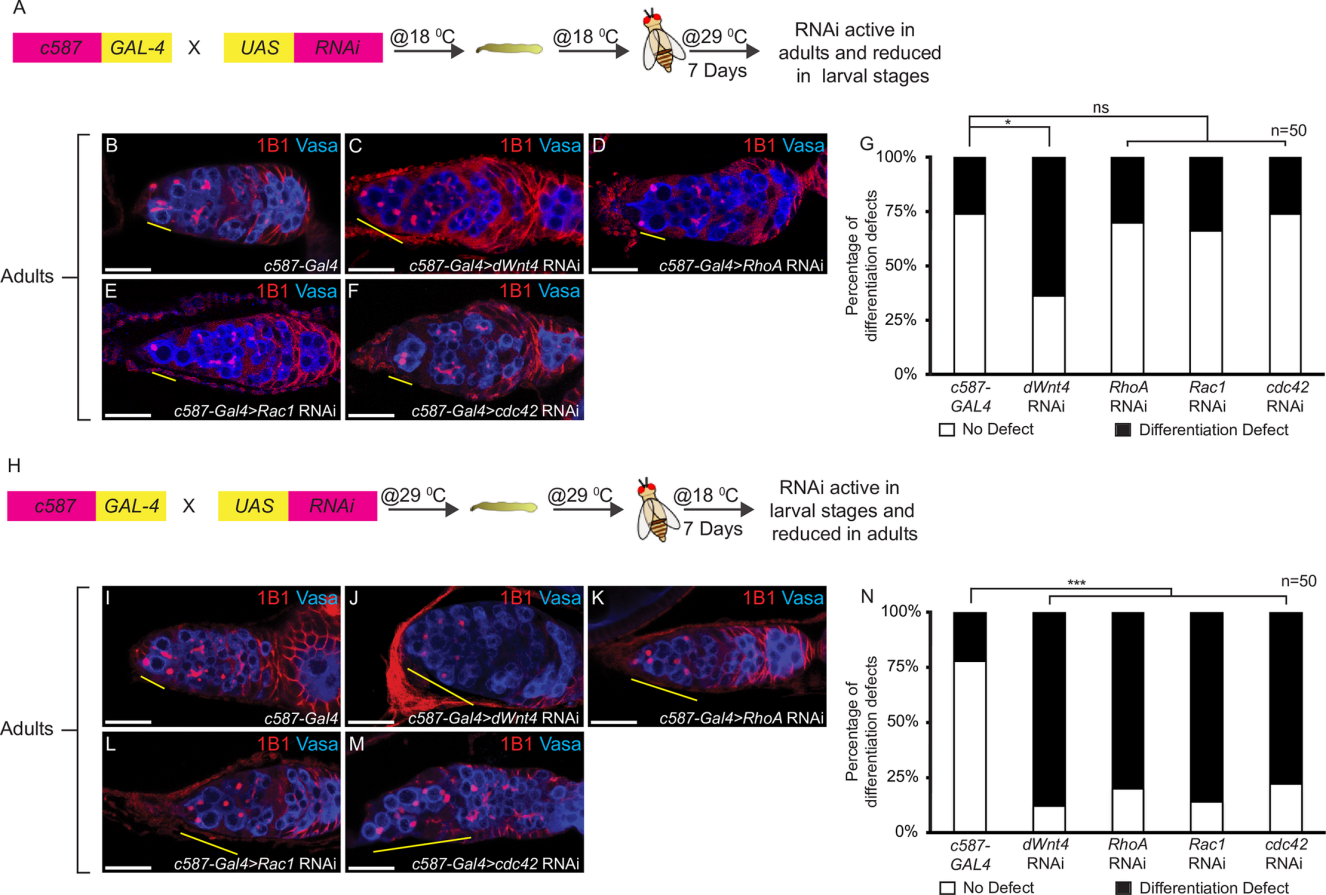
The Wnt downstream non-canonical pathway components are required in the larval gonads for proper germline stem cell differentiation. (A) Illustration of 18 ^o^C to 29 ^o^C temperature shift experimental strategy. (B-F) Germaria of *c587-GAL4* (control), *dWnt4*, *RhoA*, *Rac1* and *cdc42* depleted escort cells stained with 1B1 (red) and Vasa (blue) showing an accumulation of >3 undifferentiated cells in *dWnt4* mutants (yellow line) and no accumulation in *RhoA*, *Rac1* and *cdc42* mutants. (G) Percentage of the germaria with >3 spectrosomes in 18 ^o^C to 29 ^o^C temperature shift flies in *c587-GAL4*, *dWnt4*, *RhoA*, *Rac1* and *cdc42* depleted escort cells showing a significant difference between *c587-GAL4* and *dWnt4* RNAi mutants, but not between *c587-GAL4* and *RhoA*, *Rac1* and *cdc42* mutants (n = 50). (H) Illustration of 29 ^o^C to 18 ^o^C temperature shift experimental strategy. (I-M) Germaria of *c587-GAL4* (control), *dWnt4*, *RhoA*, *Rac1* and *cdc42* depleted escort cells stained with 1B1 (red) and Vasa (blue) showing an accumulation of >3 undifferentiated cells in *dWnt4*, *RhoA*, *Rac1* and *cdc42* depleted escort cells (yellow line). (N) Percentage of the germaria with >3 spectrosomes in 29 ^o^C to 18 ^o^C temperature shift flies, in *c587-GAL4*, *dWnt4*, *RhoA*, *Rac1* and *cdc42* depleted escort cells showing a significant difference between *c587-GAL4* and *dWnt4*, *RhoA*, *Rac1* and *cdc42* depleted escort cells (n = 50). Scale bar for all images is 20μm.

To determine if the different expression patterns of the downstream Wnt non-canonical pathway components mirrored their role in the two developmental stages, we used the UAS-GAL4 system to deplete genes using RNAi in the escort cells at specific developmental time points. The UAS-GAL4 system is temperature dependent and therefore, different temperatures can be used to attenuate genes at various developmental time points. Maximal RNAi activity is attained at 29 ^o^C whereas minimal RNAi activity is attained at 18 ^o^C [113]. c587 is expressed in the larval gonad, including the ICs, but is only expressed in the escort cells of the adult inner germarium **(S8A–S8C1 Fig)**. To elucidate if the downstream Wnt non-canonical pathway components are required in the adults to regulate CB differentiation, the flies were kept at 18 ^o^C until they eclosed. Once eclosed, the flies were shifted to 29 ^o^C and kept at this temperature for 7 days [114] **(Fig 4A)**. The germaria were then stained for 1B1 and Vasa. This strategy has been previously used to show that the dWnt4 canonical pathway is required in the adults to regulate CB differentiation [45]. We depleted *dWnt4*, *RhoA*, *Rac1*, *cdc42* and also expressed *cdc42*^*DN*^ in the escort cells to assay for differentiation defects. We found that compared to control, 18 ^o^C-29 ^o^C temperature shift *dWnt4* mutants exhibited a differentiation defect **(Fig 4B and 4C and Fig 4G)**. In contrast, compared to control, 18 ^o^C-29 ^o^C temperature shift *RhoA*, *Rac1*, *cdc42* and *cdc42*^*DN*^ mutants did not exhibit a significant accumulation of undifferentiated cells, suggesting that the downstream Wnt non-canonical pathway components do not play a critical role in the adult to regulate CB differentiation (3 ± 1 for *c587-GAL4*; 4 ± 1 for *c587-GAL4>dWnt4* RNAi, P-value = 0.002958; 3 ± 2 for *c587-GAL4>RhoA* RNAi, P-value = 0.431434; 3 ± 1 for *c587-GAL4>Rac1* RNAi, P-value = 0.91346, 3 ± 1 for *c587-GAL4>cdc42* RNAi, P-value = 0.54991 and 3 ± 1 for *c587-GAL4>cdc42*^*DN*^, P-value = 0.242545 {for all n = 50}) **(Fig 4B and Fig 4D–4G) (S9A and S9B Fig, S9E–S9G Fig)**. To elucidate if the downstream Wnt non-canonical pathway components are required in the larval gonad to regulate CB differentiation, the flies were kept at 29 ^o^C until they eclosed. Once eclosed, the flies were shifted to 18 ^o^C and kept at this temperature for 7 days [114] **(Fig 4H).** Compared to control, 29 ^o^C-18 ^o^C temperature shift *dWnt4*, *RhoA*, *Rac1*, *cdc42* and *cdc42*^*DN*^ mutants resulted in an accumulation of undifferentiated cells (2.5 ± 1 for *c587-GAL4*; 5.6 ± 2 for *c587-GAL4>dWnt4* RNAi, P-value = 1.04811E-17; 5 ± 1.5 for *c587-GAL4>RhoA* RNAi, P-value = 1.43965E-15; 5 ± 1.5 for *c587-GAL4>Rac1* RNAi, P-value = 1.53586E-18; 5 ± 2.5 for *c587-GAL4>cdc42* RNAi, P-value = 2.34120E-18 and 5 ± 1 for *c587-GAL4>cdc42*^*DN*^, P-value = 1.49752E-17{for all n = 50}) **(Fig 4I–4N) (S9C–S9F and S9H Fig)**. Taken together, these results demonstrate that the Wnt non-canonical pathway components act primarily prior to the adult stage to regulate CB differentiation.

**Fig 5:**
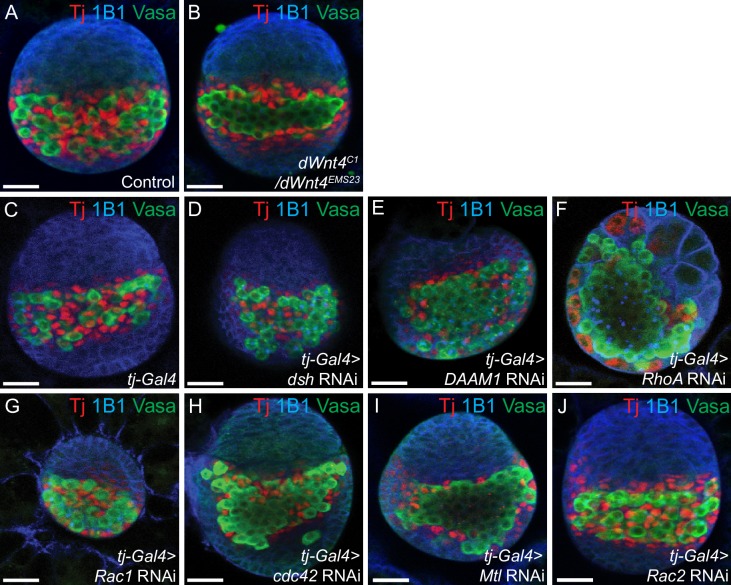
*dWnt4* and downstream Wnt non-canonical components act in the larval gonad to regulate the intermingling of ICs. (A-J) Larval gonads of control, *dWnt4* mutant, *tj-GAL4* (control), *dsh*, *DAAM1*, *RhoA*, *Rac1*, *cdc42*, *Mtl*, and *Rac2* depleted ICs stained with Tj (red), 1B1 (blue) and Vasa (green) showing loss of intermingling of ICs in *dWnt4* mutant, *dsh*, *DAAM1*, *RhoA*, *Rac1 cdc42* and *Mtl* mutants (n = 25). *Rac1* depletion results in small gonads. No intermingling defect was observed in *Rac2* mutants (n = 25). Scale bar for all images is 20μm.

As *RhoA*, *Rac1* and *cdc42* are highly expressed and active in the larval gonad and were not required in the adult germaria for CB differentiation, we asked if these proteins are required earlier in development. To test this we used an IC driver *traffic jam*-*GAL4 (tj-GAL4)*. Tj is expressed only in ICs in the late larval gonad, therefore *tj-GAL4* is a more restricted driver for ICs [11,29]. We depleted *dsh*, *DAAM1*, *RhoA*, *Rac1*, *cdc42* and expressed *RhoA*^*DN*^ and *cdc42*^*DN*^ versions in the ICs, in the late larval stages using *tj*-*GAL4*. Because we posited that RhoA, Rac1 and cdc42 act downstream of dWnt4, we also monitored the late larval gonads of *dWnt4* mutants for defects, by staining for Vasa, Tj and 1B1. We found that compared to *dWnt4* heterozygotes, *dWnt4* mutants and *dsh*, *DAAM1*, *RhoA*, *RhoA*^*DN*^, *Rac1*, *cdc42* and *cdc42*^*DN*^ mutants showed loss of intermingling of ICs with PGCs (n = 25) **(Fig 5A–5H) (S10A–S10C Fig)**. Although loss of *Rac1* in ICs led to loss of intermingling, the phenotype was weaker than *dWnt4* mutants, *RhoA* and *cdc42* depleted ICs. Additionally, depletion of *Rac1* in the ICs resulted in a decrease in the size of the larval gonad. It has been shown *Rac1*, *Ras-related C3 botulinum toxin substrate 2* (*Rac2)* and *Mig-2-like (Mtl)* act redundantly to regulate PCP [115]. For this reason, we also depleted *Mtl* and *Rac2* specifically in the ICs and found that *Mtl* depleted ICs resulted in a strong loss of ICs intermingling but *Rac2* depleted ICs did not exhibit any intermingling defect **(Fig 5I and 5J)**. Although we find that depletion of *Rac2* does not lead to a differentiation defect, we do not know if this is due to a feeble RNAi mediated depletion, or in fact *Rac2* does not play a role. These results suggest that *dsh*, *DAAM1*, *RhoA*, *Rac1*, *Mtl* and *cdc42* regulate intermingling of ICs in the larval gonad.

In order to further validate our results obtained by RNAi and expression of dominant negative forms of *RhoA* and *cdc42*, we also analyzed larval gonads of *dsh*^*1*^ mutants, *cdc42*^*5*^, a hypomorphic allele that has been previously shown to exhibit mild PCP defects, *cdc42 (cdc42*^*2*^*/cdc42*^*5*^*)* hypomorphic mutant and heterozygous larval gonads of flies having one genetically reduced copy of all three *Rac* genes [115]. We found that these mutants and the heterozygous larval gonad for flies that remove all three *Rac* genes also exhibited loss of ICs intermingling (69% *dsh*^*1*^ mutants, n = 20; 55% *cdc42*^*5*^ mutants, n = 20; 80% *cdc42 (cdc42*^*2*^*/cdc42*^*5*^*)* mutants, n = 20 and 60% heterozygous *Rac1*, *Rac2* and *Mtl* larval gonads, n = 10) **(S10D–S10H Fig)**. Together, these results suggest that *RhoA*, *Rac* genes (*Rac1* and *Mtl*) and *cdc42* regulate intermingling of ICs.

### Components of the Wnt non-canonical pathway regulate IC number

As *RhoA*, *Rac1* and *cdc42* are known to play a role in cell division, we wondered if there were additional defects apart from loss of intermingling in these mutants. We found that compared to *dWnt4* heterozygotes and *tj-GAL4*, *dWnt4* mutants and *RhoA*, *Rac1*, *Mtl* and *cdc42* mutant larval gonads had significantly fewer ICs (151 ± 18 for *dWnt4*/CyO; 84 ± 8 for *dWnt4* mutants, P-value = 6.74402E-05; 213 ± 36 for *tj-GAL4*; 36 ± 13 for *tj-GAL4>RhoA* RNAi, P-value = 5.16854E-06; 67 ± 14 for *tj-GAL4>Rac1* RNAi, P-value = 9.83637E-05; 111 ± 10 for *tj-GAL4>Mtl* RNAi, P-value = 0.000231 and 61 ± 17 for *tj-GAL4>cdc42* RNAi, P-value = 2.07541E-05 {for all n = 5}). It is possible that the decrease in the number of ICs is either due to decrease in their division rate or due to death of ICs. To test if loss of *dWnt4*, *RhoA*, *Rac1*, *Mtl* and *cdc42* affects the division rate of the ICs, we stained these mutants with a mitotic marker, Phospho-Histone 3 (PH3), Tj and 1B1. We observed that *RhoA*, *Rac1*, *Mtl* and *cdc42* depleted IC mutants show a decrease in the number of ICs expressing PH3, suggesting that *RhoA*, *Rac1*, *Mtl* and *cdc42* also regulate division of the ICs. However, *dWnt4* mutants did not exhibit this defect compared to heterozygous, but displayed a defect compared to wild type (WT) control (12.5 ± 2 for WT control; 4.5 ± 2 for *dWnt4*/CyO, P-value = 1.72943E-08; 3 ± 2 for *dWnt4* mutants, P-value (WT) = 7.42348E-09, P-value (*dWnt4*/CyO) = 0.104558; 9 ± 3 for *tj-GAL4*, n = 10; 4 ± 2 for *tj-GAL4>RhoA* RNAi, n = 10, P-value = 0.00010; 3 ± 2 for *tj-GAL4>Rac1* RNAi, n = 10, P-value = 2.56584E-05; 7 ± 2 for *tj-GAL4>Mtl* RNAi, n = 10, P-value = 0.035212, and 5 ± 3 for *tj-GAL4>cdc42* RNAi, n = 10, P-value = 0.003773) **(S11A–S11H Fig)**. To determine if the ICs in these mutants show increased cell death, we stained them for a death marker, cleaved Caspase3, along with Tj and 1B1. We observed that only ICs of *RhoA* mutants showed Caspase3 staining. Compared to 0% of Tj positive cells in control, 3% of Tj positive cells in *RhoA* depleted IC mutants exhibited Caspase3 staining indicating that in addition to its role in cell division, *RhoA* also regulates IC survival (n = 5) **(S11I–S11O Fig)**. Together, these results show that *dWnt4* and the downstream components of the Wnt non-canonical pathway, *RhoA*, *Rac1*, *Mtl* and *cdc42* play a critical role to regulate intermingling and cell division of ICs in the larval gonad. Additionally, as *RhoA* depletion exhibited a stronger defect than *dWnt4* mutants it is likely that it has roles independent of dWnt4 signaling.

*dWnt4* mutants and *RhoA*, *Rac1* and *cdc42* depleted escort cell germaria exhibit reduced number of escort cells and loss of CB encapsulation [46]. Additionally, depletion of *RhoA*, *Rac1* and *cdc42* in the ICs resulted in reduced number of ICs. We asked if reduction in the escort cell number and loss of encapsulation of CB in the adults is a consequence of reduced number of ICs in the larval gonad. To answer this question, we counted the number of Tj positive escort cells in the adult flies, of 29 ^o^C-18 ^o^C temperature shift escort cell depleted *RhoA*, *Rac1* and *cdc42* mutants. We found that these mutants exhibited significantly lower number of escort cells (15.4 ± 2.6 for *c587-GAL4*; 10.4 ± 2.6 for *c587-GAL4>RhoA* RNAi, P-value = 0.00048; 10.6 ± 2 for *c587-GAL4>Rac1* RNAi, P-value = 0.00068 and 6.9 ± 1.6 for *c587-GAL4>cdc42* RNAi, P-value = 8.1045E-08 {for all n = 10}). These results suggest that *RhoA*, *Rac1* and *cdc42* act in the ICs to regulate the escort cell number and therefore, proper GSC differentiation in the adults.

### Wnt canonical pathway and PCP regulators do not regulate the intermingling of ICs in the larval gonad

To determine if PCP core components regulate intermingling in the larval gonad we depleted *vang/stbm*, *ds*, *fz and ft* in ICs and found that depletion of these genes did not exhibit any intermingling defects **(S12A–S12E Fig)**. To ascertain that the canonical pathway is not required in the larval gonad, we depleted the canonical specific co-receptor, *arr* in ICs and found that these mutants also did not exhibit any intermingling defect **(S12A and S12F Fig).** We used *arr* as *β-catenin* has roles outside of Wnt signaling and loss of *arr* in the adult escort cells results in CB differentiation defects [45,116]. These results suggest that the Wnt canonical pathway and *vang/stbm*, *ds*, *fz* and *ft*, the core components and Ds/Ft systems that define PCP, do not play a critical role in the ICs.

### dWnt4 regulates RhoA, Rac and cdc42 activity in the larval gonad

**Fig 6:**
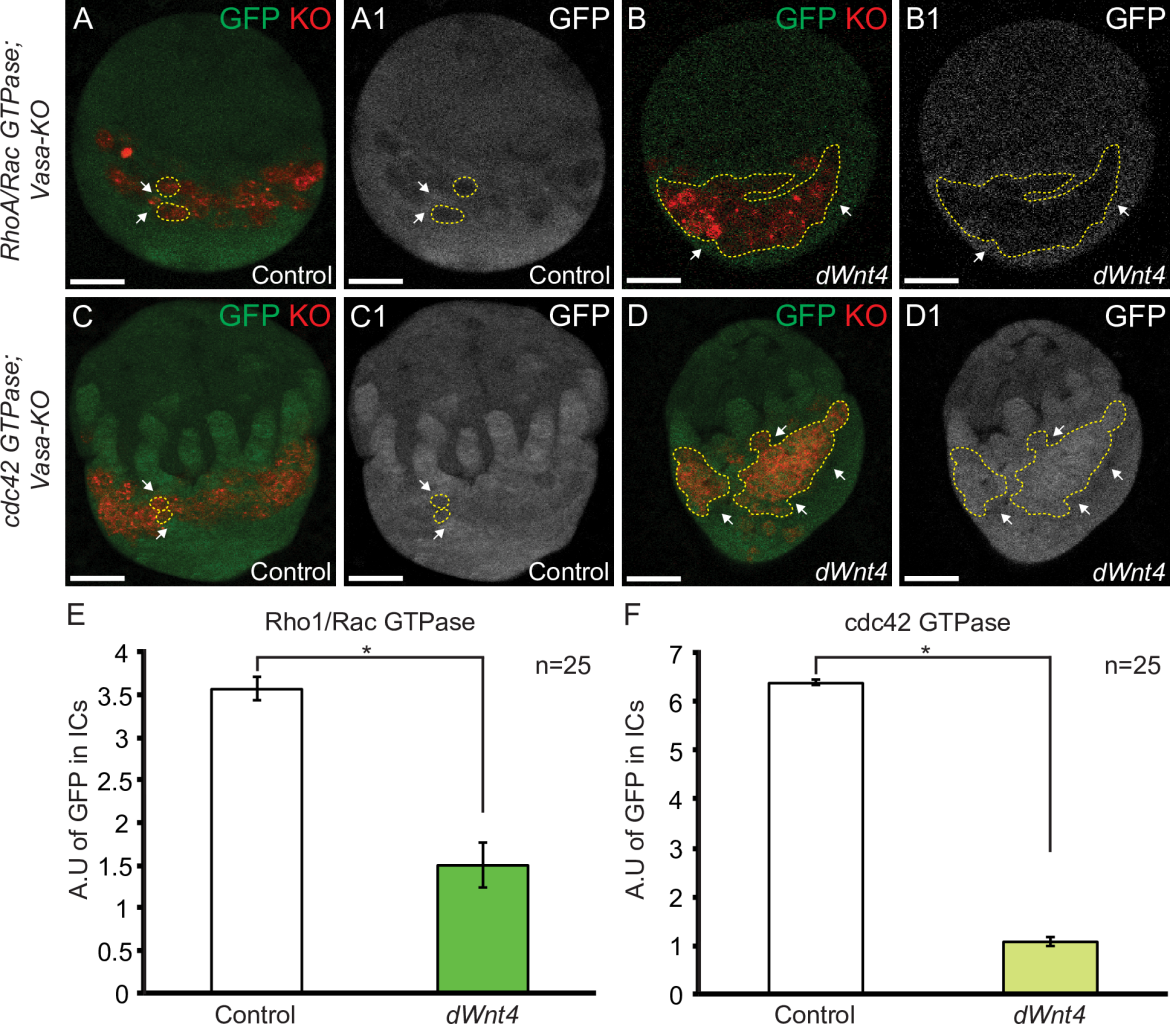
*dWnt4* modulates the activity of downstream Wnt non-canonical pathway components in the larval gonad. (A-D1) Larval gonad of control and *dWnt4* mutants showing downregulation of activated RhoA/Rac and cdc42 in the ICs of *dWnt4* mutants (white arrows). GFP channel is shown in A1, B1, C1 and D1. A single PGC (control) and loss of intermingling (*dWnt4* mutants) are highlighted by dashed yellow line. (E-F) Quantification of GFP in the ICs showing downregulation of activity of RhoA/Rac and cdc42 reporters in *dWnt4* mutants. Scale bar for all images is 20μm.

Similar to *dWnt4* mutants, *RhoA*, *Rac1* and *cdc42* depletion in the ICs showed a defect in intermingling of ICs. To determine if *RhoA*, *Rac1* and *cdc42* act downstream of *dWnt4*, we analyzed the expression and activity of RhoA, Rac1 and cdc42 with the help of the GFP tagged lines, in larval gonads of both control and *dWnt4* mutants. We found that compared to the control, expression of RhoA, Rac1 and cdc42 was not altered in the ICs of *dWnt4* mutants, suggesting that dWnt4 does not regulate the protein levels of RhoA, Rac1 and cdc42 (P-value = 0.64182 for RhoAGFP, P-value = 0.79908 for Rac1GFP, and P-value = 0.63675 for cdc42GFP in *dWnt4* mutants {for all n = 3}) **(S13A–S13G Fig)**. The activity level of RhoA/Rac and cdc42 was determined using biosensors in *dWnt4* mutants. To ensure that there is no background, we also imaged larval gonads, without the biosensors (**S13H–S13I1 Fig)**. We found that RhoA/Rac and cdc42 activity was significantly downregulated in ICs (P-value = 0.02067, n = 25 for RhoA/Rac activity and P-value = 0.01369, n = 25 for cdc42 activity) **(Fig 6A–6F).** We also observed cdc42 activity in the terminal filaments and found that it was not altered in *dWnt4* mutants (P-value = 0.35901, n = 3) **(S13J Fig).** These results suggest that *dWnt4* regulates the activity of RhoA, Rac and cdc42 in the ICs to regulate intermingling of ICs with PGCs.

## Discussion

**Fig 7:**
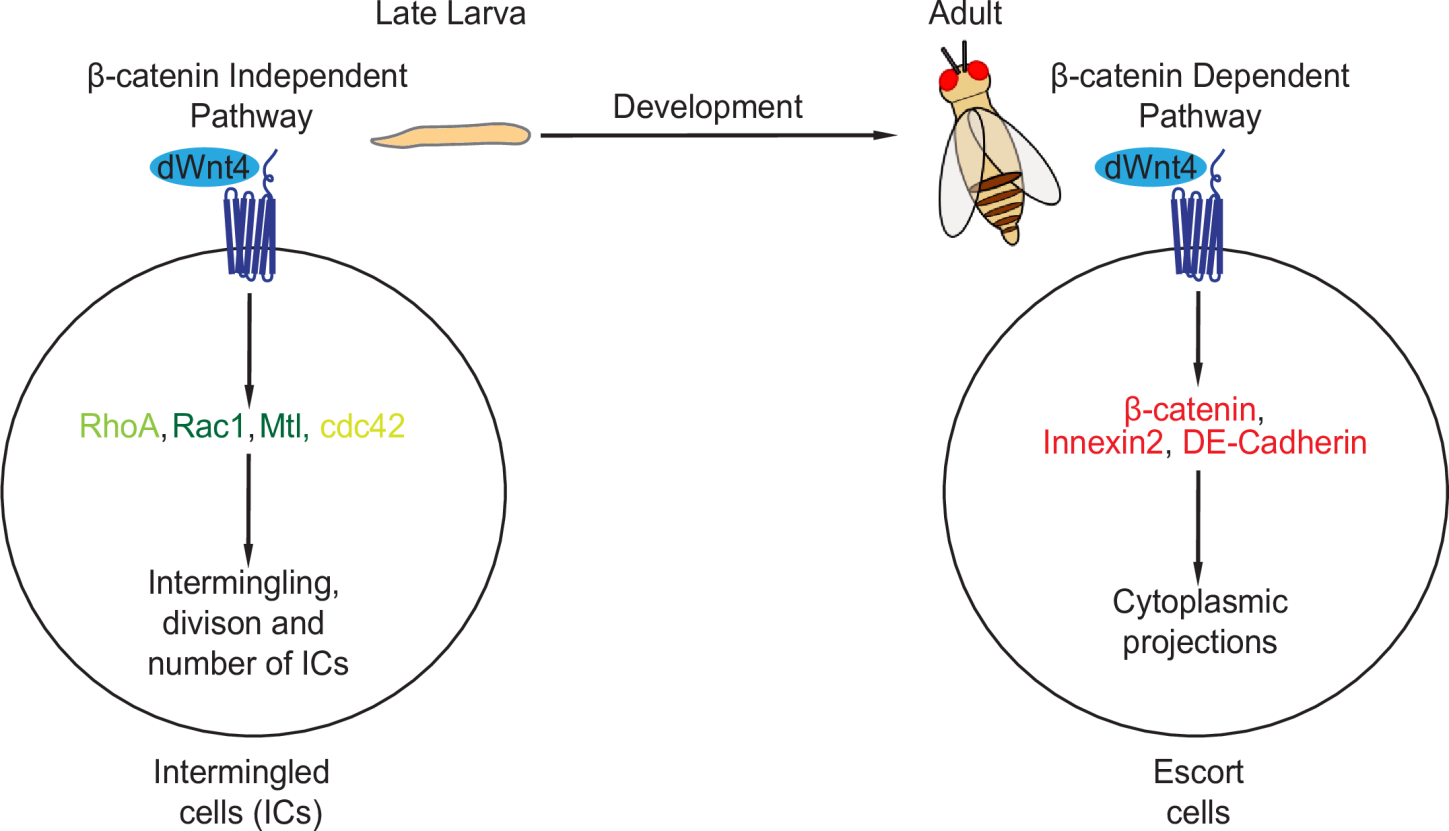
dWnt4 switches from a β-catenin independent pathway to a β-catenin dependent pathway during *Drosophila* oogenesis to regulate the formation of GSC differentiation niche. A schematic showing that dWnt4 uses both β-catenin independent and β-catenin dependent pathways to regulate GSC differentiation. In the larval gonad, dWnt4 uses RhoA, Rac1, Mtl and cdc42 to regulate intermingling, division and number of the ICs while in the adult, dWnt4 uses the β-catenin dependent pathway to regulate the cytoplasmic protrusions.

Here, we find that *dWnt4* regulates CB differentiation through the downstream non-canonical pathway components. We show that RhoA, Rac1 and cdc42 are expressed at high levels in the ICs of the larval gonad and are active while the expression of Fz3RFP, a β-catenin-dependent transcriptional reporter, is not detectable. Conversely, we find low levels of RhoA, Rac1 and cdc42 in the adults but the β-catenin-dependent canonical reporter is expressed at high levels. Consistent with this, we find a role for RhoA, Rac1 and cdc42 but not the β-catenin-dependent canonical pathway in the larval gonad for promoting intermingling of ICs **(Fig 7)**. Our results, in conjunction with Mottier-Pavie *et al*. and Wang *et al*., that show the canonical pathway is required in the adult, point towards a switch from utilizing Wnt non-canonical components to utilizing a canonical pathway to regulate the formation of the differentiation niche [44,45].

Our findings that the depletion of *vang/stbm*, *ds*, *fz* and *ft* in either ICs or escort cells does not lead to differentiation defects, suggests that PCP does not play a critical role in regulating CB differentiation. These results are consistent with previous literature, where Cohen *et al*. showed that mutations in, *fz*, *fmi*, *dgo*, *vang/stbm* and *pk*, genes required for PCP of a cell, do not lead to significant disruption of ovarian morphology [82]. We show that the other PCP system components, *ds* and *ft*, are also not required to regulate differentiation. Together, Cohen *et al*. and our results suggest that it is not the PCP system, but the downstream Wnt non-canonical components that play a critical role in the differentiation niche to regulate differentiation. We propose that *dWnt4* uses the downstream Wnt/Fz non-canonical pathway to regulate formation of the differentiation niche.

We find that *RhoA*, *Rac1* and *cdc42* regulate intermingling of ICs in the larval gonad. In addition, we find that loss of *RhoA* leads to death of ICs and loss of *Rac1* leads to decrease in the larval gonad size. *dWnt4* mutants do not show either of these outcomes. Together, these results suggest that *RhoA* and *Rac1* have roles in the larval gonad independent of Wnt signaling. It has been extensively shown that Rac1, Rac2 and Mtl have redundant roles in axon growth, guidance and PCP in the eye and the wing [93,95,115]. Indeed, we found that *Rac1* depleted ICs resulted in loss of intermingling but this phenotype was not as strong as *dWnt4*, *RhoA* and *cdc42* depleted ICs. *Rac2* depleted ICs resulted in no intermingling defect. However, depletion of *Mtl* in the ICs resulted in a strong loss of intermingling phenotype. This suggests that small GTPases play a critical role in ICs function, both independently and in coordination with Wnt signaling.

In the adult, escort cells extend dynamic cytoplasmic protrusions to encapsulate the GSCs, the differentiating daughter and differentiating cysts, a function necessary for proper differentiation [28,39–41,102,117]. It was recently shown that in adult female germaria, *stat* regulates the formation of these protrusions through cdc42. Loss of *stat* or *woc*, a component of the stat pathway, leads to formation of smaller protrusions instead of longer stable protrusions. Loss of *woc* also leads to an accumulation of undifferentiated cells. Overexpression of *cdc42* in *woc* mutants rescued the small protrusion defect and partially rescued the phenotype. However, Banisch *et al* report that expression of dominant active or dominant negative *cdc42* in the escort cells only mildly affects protrusions and does not lead to an accumulation of undifferentiated cells. Our temperature shift experiments also suggest that expression of dominant negative *cdc42* exclusively in the adults does not result in an accumulation of undifferentiated cells, while its expression prior to eclosion does **(S9C and S9D and S9E and S9F Fig)**. Moreover, Banisch *et al*. also show that active cdc42 is expressed in the escort cells and active RhoA could not be detected in most of the escort cells using Dia-RBD:GFP but could be detected mainly in the escort cell body and weakly in the escort cell protrusion using Capu-RBD:GFP [117]. We think that *cdc42* may have a role in the adults by controlling protrusive activity of escort cells, but it does not affect CB differentiation in a biologically meaningful way. This suggests that cdc42 and Rho activity in the adult is attenuated and their activity is independent of dWnt4.

Cell movement requires dynamic actin-myosin polymerization at the leading edge, driven by Rac1 and cdc42, and actin-myosin contraction at the lagging end, driven by RhoA [118,119]. In our study, we find that both RhoA/Rac and cdc42 are present and active in the ICs. Loss of *RhoA*, *Rac1* and *cdc42* leads to loss of intermingling of ICs, suggesting that these proteins drive ICs cell movement and intermingling in the larval gonad. In contrast, only cdc42, but not RhoA, is active in the adult escort cells, consistent with the fact that these cells are stationary [41,117]. Thus the switch in the module used by Wnt signaling to regulate the activity of RhoA/Rac and cdc42 parallels the developmental demands of these cells to first move between germ cells in the larval gonad, then to stop and create long, stable protrusions that promote differentiation in the pupal and adult gonad.

Cancer metastasis requires cell migration. The invasion of metastatic cancer cells requires cells to lose epithelial properties and gain mesenchymal properties. During this process, known as Epithelial-mesenchymal transition (EMT), the epithelial cells, which are otherwise polarized, non-motile and have strong cell-cell interaction, subsequently lose polarity, cell-cell adhesion and become motile [120,121]. *RhoA*, *Rac1* and *cdc42* are conserved from lower eukaryotes to mammals and are key players that affect cell division, cytoskeletal rearrangement, cell polarity, and cell motility [76,77,79,80,91,106,118]. It has been previously discovered that during cancer, these proteins are upregulated and therefore help initiate metastasis [122]. We have discovered that RhoA, Rac1 and cdc42 are turned on in the ICs of the larval gonad and switched off in the adult escort cells. This is fascinating because the ICs and the escort cells are essentially the same cells at different developmental time points. A better understanding of how this switch is mediated may give us an insight into cancer and aid in developing mechanisms to block metastasis.

## Materials and methods

### Fly stocks

The following fly stocks were used in the study: *c587-Gal4*, *trafficJam (tj)-Gal4*, *dWnt4*^*C1*^*/*CyO (6651), *bamGFP*, *arrow* RNAi (31313), *Rho1* RNAi (Bloomington 32383), *UASRho*^*DN*^ (Bloomington 7327), *Rac1* RNAi (Bloomington 28985 and 34910), *Mtl* RNAi (Bloomington 51932), *Rac2* RNAi (v28926), *cdc42* RNAi (Bloomington 35756 and 37477), *UAScdc42*^*DN*^ (Bloomington 6288), *dDAAM1* RNAi (Bloomington 39058, V24885), *dsh* RNAi (v101525), *frizzled* RNAi (Bloomington 34321), *van gogh* RNAi (Bloomington v7376), *daschous* RNAi (Bloomington 28008), *fat* RNAi (Bloomington 29566), *y*^*1*^ w^*^; *Rho1^72F^**/*CyO (Bloomington 7326), *y*^*1*^ *w67c23*; *P{EPgy2}Rac1^EY05848^*/TM6B, Tb^1^ (Bloomington 15461), y[1] w[*]; Rac1[J10] Rac2[Delta] P{w[+mW.*hs] = FRT(w[hs])}2A Mtl[Delta]/TM6B*, *Tb[1]* (Bloomington 6679), *y1* w^*^ *Cdc42^3^*/FM6 (Bloomington 7337), *y*^*1*^ w^*^ *Cdc42*^*2*^ *P{neoFRT}19A* (Bloomington 9105), *y*^*1*^ w^*^ *Cdc42*^*5*^ *P{neoFRT}19A* (Bloomington 52237), *y*^*1*^ *Mi{MIC}DAAM^MI04569^* *w1118*/FM7h (Bloomington 38567), *w*^*1*^ *dsh^1^* (Bloomington 5298), RhoAGFP (V318439), Rac1GFP (Bloomington 52285), cdc42GFP (V218151), w*;P{sqh-Pkn.RBD.G58A-eGFP}312a P{sqh-Pkn.RBD.G58A-eGFP}312b (Bloomington 52298), w*;P{sqh-WASp.RBD-GFP}378a P{sqh-WASp.RBD-GFP}378b (Bloomington 56746), *UASmCD8GFP* (Bloomington 32184), *faxGFP*, *Sco/*CyO;MKRS/TM6 (Lehmann Lab), *Sco*/CyO;*Nos-Gal4*::*VP16*,*Vasa-KO/*TM6 (Lehmann Lab); *Sco/*Cyo;*Fz3RFP* (Bach Lab).

### Collection and Fixation of tissues

3–4 day old fly ovaries were dissected in PBS, fixed for 30 min in PBS plus 5% formaldehyde, incubated for 1 h in PBST (0.2% Tween 20 (Sigma) in PBS) supplemented with 1% Triton X-100 (Sigma), followed by incubation for 2 h in BBT (PBST supplemented with 1% (w/v) bovine serum albumin (BSA; Sigma)). Primary antibodies were added in BBT and incubation was carried out overnight at 4 ^o^C. The following day, ovaries were washed four times for 10 min, 20 min, 30 min in BBT and for 30 min in BBT supplemented with 2% (w/v) donkey serum (Sigma). Secondary antibodies were added in BBT supplemented with 4% (w/v) donkey serum (Sigma) and incubated for 2 h followed by five washes, 10 min each in PBST. VECTASHIELD (Vector Laboratories) with DAPI was added prior to mounting. Fixation and staining of larval gonads was carried out as previously described [123].

### Temperature shift experiment

In order to express a specific gene only in the adults, the flies were kept at 18 ^o^C until they eclosed. Once eclosed, the young flies were shifted to 29 ^o^C and kept at this temperature for 7 days. Staining was followed to determine any differentiation defects.

In order to express a specific transgene only in the larval stage, the flies were kept at 29 ^o^C until they eclosed. Once eclosed, the young flies were shifted to 18 ^o^C and kept at this temperature for 7 days. These flies were then dissected and stained for observation.

Unless specified, all experiments that utilized the UAS-GAL4 system were performed constitutively at 29 ^o^C.

### Antibodies

Immunostaining of the ovaries and larval gonad was carried out with the following Primary antibodies: Mo 1B1 (1:20, DSHB), Rb Vasa (1:5000, Rangan Lab), Ch Vasa (1:500, Rangan Lab), GP Traffic Jam (1:5000, Godt Lab), Rb GFP (1:2000, ab6556), Rb pMAD (1:200, abcam AB52903), Mo BamC (1:200, DSHB), Rb Bruno (1:500, Lehmann Lab), Rb PH3 (1:200, Cell Signaling 97015), and Rb Caspase 3 (1:300, Cell Signaling 96615).

Alexa 488 (Molecular Probes), Cy3 and Cy5 (Jackson Labs) conjugated secondary antibodies were used at a concentration of 1:500.

### Fluorescence imaging

The tissues were visualized under 10X, 20X, 40X and 63x objective lenses. The images were acquired using a Zeiss LSM-710 confocal microscope under 20x, 40X and 63x objective.

### Live imaging

The tissues were dissected in Schneider’s media and mounted. These were then visualized under 20X objective lens. The images were acquired using a Zeiss LSM-710 confocal microscope under 20x objective.

### Quantification analysis

#### A.U of protein levels

In order to calculate intensities for Fz3RFP and GFP in ICs or escort cells, images for both, control and mutant larval gonad and adult germaria were taken using the same confocal settings. Z stack planes were obtained for all images. Specific planes showing Tj positive ICs or escort cells were chosen, the area of Tj positive cell was outlined and analyzed using the ‘analyze’ tool in ImageJ. The mean and area of the specified region was obtained. An average of all the ratios (Mean/Area), for Fz3RFP, GFP and Tj, per image was calculated for both, control and mutants. The average ratio for Fz3RFP and GFP was normalized with average ratio for Tj. These are the arbitrary units (A.U) for Tj, GFP and RFP for this region. A minimum of 7 germaria and 7 larval gonads (minimum 5 Tj positive cells per image) were considered for the each quantification.

#### A.U of activity reporters

In order to calculate intensities of activity reporters, images with Z stacks were obtained for both, control and mutant larval gonads using the same confocal settings. For ICs, an area right besides a PGC was outlined and for terminal filament an area in the terminal filament was outlined. All these areas were analyzed using the ‘analyze’ tool in ImageJ. The mean and area of the specified region was obtained (25 cells were analyzed per image). An average of all the ratios (Mean/Area), per image was calculated for control and mutants. The average ratio for activity was subtracted with average ratio for background. The results are the arbitrary units (A.U) for activity reporters for this region. 25 cells of 2 larval gonads each were quantitated for RhoA/Rac and cdc42.

In order to calculate intensities of activity reporters between larval gonads and adult germaria, A.U of GFP was normalized to Vasa-KO. 5 cells of 3 larval gonads and adult germaria each were quantitated for RhoA/Rac and cdc42.

The number of Tj and Fz3RFP positive cells were calculated manually in all stacks for 10 germaria in ImageJ.

### Statistical analysis

P-values were determined by two-tailed equal variance t test in mutants vs. wild type strains. Z-score was determined by a two-tailed test for data represented in percentage.

### Materials and reagents

Fly food was created using the procedures from the Ruth Lehmann lab at NYU (summer/winter mix), and used to fill narrow vials to approximately 12mL.

## Supporting information

S1 Fig Planar cell polarity (PCP) regulators do not play a critical role in the formation of the differentiation niche. (A) Quantification of spectrosomes in *c587-GAL4*, *dsh*, *DAAM1*, *RhoA*, *Rac1* and *cdc42* depleted escort cells showing a significant difference in mutants (n = 50). (B) A schematic showing proteins that regulate the PCP of a cell. (C-G) Germaria of *c587-GAL4* (control), *vang*, *ds*, *fz* and *ft* depleted escort cells stained with 1B1 (red) and Vasa (blue) showing no accumulation of undifferentiated cells in *vang*, *ds*, *fz* and *ft* depleted escort cells (yellow line). (H) Percentage of the germaria with >3 spectrosomes in *c587-GAL4*, *vang*, *ds*, *fz* and *ft* depleted escort cells showing no difference in *vang*, *ds*, *fz* and *ft* depleted escort cells (n = 50). (I) Quantification of spectrosomes in *c587-GAL4*, *vang*, *ds*, *fz and ft* depleted escort cells showing no significant difference (n = 50). Scale bar for all images is 20μm.
(TIF)

S2 Fig The Wnt downstream non-canonical pathway components are required in the escort cells for proper germline stem cell differentiation. (A-C) *dsh*^*1*^*/+*, *RhoA/*CyO, and *Rac1*/TM6 stained with 1B1 (red) and Vasa (blue) showing no accumulation of undifferentiated cells (yellow line). (D) Percentage of the germaria with >3 spectrosomes in *dsh*^*1*^*/+*, *RhoA/*CyO, *Rac1*/TM6, *dsh*^*1*^ mutants, *dsh*^*1*^/*RhoA* trans-heterozygote and *dsh*^*1*^/*Rac1* trans-heterozygote showing a significant difference in differentiation defects in the trans-heterozygotes (n = 50). (E) Quantification of the number of spectrosomes in *dsh*^*1*^*/+*, *RhoA/*CyO, *Rac1*/TM6, *dsh*^*1*^ mutants, *dsh*^*1*^/*RhoA* trans-heterozygote and *dsh*^*1*^/*Rac1* trans-heterozygote showing a significant difference in the number of spectrosomes in the trans-heterozygotes (n = 50). (F-H) *dsh*^*1*^ mutants, *dsh*^*1*^/*RhoA* trans-heterozygote and *dsh*^*1*^/*Rac1* trans-heterozygote stained with 1B1 (red) and Vasa (blue) showing an accumulation of >3 undifferentiated cells in the trans-heterozygotes (yellow line). Scale bar for all images is 20μm.
(TIF)

S3 Fig The Wnt downstream non-canonical pathway components are required in the escort cells for proper cystoblast differentiation. (A-D2) Germaria of *c587-GAL4* (control), *RhoA*, *Rac1* and *cdc42* depleted escort cells stained with pMAD (red), GFP (green) and Vasa (blue) showing an accumulation of >3 pMAD and Bam negative cells in *RhoA*, *Rac1* and *cdc42* mutants (yellow line). pMAD channel is shown in A1, B1, C1 and D1; GFP channel is shown in A2, B2, C2 and D2. (E) Quantification of number of pMAD and Bam negative cells in *c587-GAL4*, *RhoA*, *Rac1* and *cdc42* depleted escort cells showing a significant increase in pMAD and Bam negative cells in *RhoA*, *Rac1* and *cdc42* mutants (n = 20). Scale bar for all images is 20μm.
(TIF)

S4 Fig The Wnt downstream non-canonical pathway components are required in the escort cells for proper BamC expression. (A-D2) Germaria of *c587-GAL4* (control), *RhoA*, *Rac1* and *cdc42* depleted escort cells stained with pMAD (red), BamC (green) and Vasa (blue) showing an accumulation of >3 pMAD and BamC negative cells in *RhoA*, *Rac1* and *cdc42* mutants (yellow line). pMAD channel is shown in A1, B1, C1 and D1; BamC channel is shown in A2, B2, C2 and D2. Scale bar for all images is 20μm.
(TIF)

S5 Fig *RhoA*, *Rac1* and *cdc42* act upstream of Bam in the escort cells to regulate cystoblast differentiation. (A) *c587-GAL4* (control) carrying a *hs-bam* transgene stained with 1B1 (red) and Vasa (blue) without heat-shock. (B-E) *c587-GAL4* (control), *RhoA*, *Rac1* and *cdc42* depleted escort cell mutants carrying a *hs-bam* transgene stained with 1B1 (red) and Vasa (blue) showing differentiating cysts marked by fusomes (red) (white arrow) and a lack of undifferentiated cells post heat-shock. (F-I1) *c587-GAL4* (control), *RhoA*, *Rac1* and *cdc42* depleted escort cell mutants carrying a *hs-bam* transgene, without heat-shock stained with 1B1 (red), Bruno (green) and Vasa (blue) showing accumulation of undifferentiated cells (yellow line) marked by the presence of spectrosomes (red) and low Bruno expression in the undifferentiated cells and early cysts, while high Bruno expression in 16-cell cyst and onwards. Bruno channel is shown in F1, G1, H1 and I1. (J-M1) *c587-GAL4* (control), *RhoA*, *Rac1* and *cdc42* depleted escort cell mutants carrying a *hs-bam* transgene stained with 1B1 (red), Bruno (green) and Vasa (blue), post heat-shock showing Bruno expression in the differentiating cysts (white arrow) marked by fusomes (red) and a lack of undifferentiated cells post heat-shock. Cysts in post heat-shock *Rho* mutants showed weak Bruno staining. Bruno channel is shown in J1, K1, L1, and M1. Scale bar for all images is 20μm.
(TIF)

S6 Fig The downstream Wnt non-canonical pathway components, RhoA, Rac1, cdc42 are temporally regulated in the ICs and adult escort cells. (A-D1) Late larval gonad of transgenic flies with GFP tagged to *Rac1* and *cdc42*, respectively stained for Tj (red), Vasa (blue) and GFP (green) showing high expression of Rac1 and cdc42 in the ICs (white arrow). 63x is shown in C-C1 and D-D1. GFP channel is shown in A1, B1, C1 and D1. (E-G1) Late pupal germaria of transgenic flies with GFP tagged to *RhoA*, *Rac1* and *cdc42*, respectively stained for Tj (red), GFP (green) and Vasa (blue) showing low expression of RhoA, Rac1, and cdc42 in pupal escort cells (white arrow). GFP channel is shown in E1, F1 and G1. (H-I1) Adult germaria of transgenic flies with GFP tagged to *Rac1* and *cdc42*, respectively stained for Tj (red), GFP (green) and Vasa (blue) showing low expression of Rac1 and cdc42 in the adult escort cells (white arrow). GFP channel is shown in H1 and I1. Scale bar for C1 and D1 is 10μm. Scale bar for all other images is 20μm.
(TIF)

S7 Fig Escort cells express activated forms of cdc42 but not of RhoA/Rac. (A-B1) Adult germaria of transgenic flies that report active form of RhoA/Rac and cdc42. While RhoA/Rac is not expressed in the escort cells, it was expressed in the follicle cells (white arrow). cdc42 is active in the adult escort cells. Germline is marked by Vasa-KO. GFP channels are shown in A1 and B1. (C) Quantification (n = 3) of GFP in the ICs and adult escort cells showing that while active RhoA/Rac is not expressed in the adult escort cells, active form of cdc42 is highly expressed in the ICs in comparison to the adult escort cells. (D-D1) Late pupal germarium of fly carrying the Wnt canonical reporter, Frizzled3 (Fz3) stained for RFP (red), Tj (green) and 1B1 (blue) showing high expression of Fz3 in the pupal escort cells (white arrow). RFP channel is shown in D1. Scale bar for all images is 20μm.
(TIF)

S8 Fig c587 is expressed in ICs of the larval gonad and escort cells of the adult germaria. (A-B1) Late larval gonad of flies with *mCD8GFP* under the control of *c587* stained for Tj (red), GFP (green) and Vasa (blue) showing GFP expression in the ICs (white arrows). 63x is shown in B-B1. GFP channel is shown in A1 and B1. (C-C1) Adult germaria of flies with *mCD8GFP* under the control of *c587* stained for Tj (red), GFP (green) and Vasa (blue) showing GFP expression in the escort cells (white arrow). GFP channel is shown in C1. Scale bar for B and B1 is 10μm. Scale bar for all other images is 20μm.
(TIF)

S9 Fig *cdc42* is required prior to eclosion to regulate germline stem cell differentiation. (A-B) Germaria of *c587-GAL4* (control) and 18 ^o^C to 29 ^o^C temperature shift *cdc42* depleted escort cells using *cdc42*^*DN*^ stained with 1B1 (red) and Vasa (blue) showing no differentiation defects in *cdc42* mutants (yellow line). (C-D) Germaria of *c587-GAL4* (control) and 29 ^o^C to 18 ^o^C temperature shift *cdc42* depleted escort cells using *cdc42*^*DN*^ stained with 1B1 (red) and Vasa (blue) showing an accumulation of >3 undifferentiated cells in *cdc42* mutants (yellow line). (E) Percentage of the germaria with >3 spectrosomes in 18 ^o^C to 29 ^o^C and 29 ^o^C to 18 ^o^C temperature shift flies, in *c587-GAL4* and *cdc42*^*DN*^ mutants showing a significant difference between *c587-GAL4* and *cdc42*^*DN*^ expressed escort cells in 29 ^o^C to 18 ^o^C but not in 18 ^o^C to 29 ^o^C (n = 50). (F) Quantification of the number of spectrosomes in 18 ^o^C to 29 ^o^C and 29 ^o^C to 18 ^o^C temperature shift flies in *c587-GAL4*, *cdc42*^*DN*^ expressed escort cells showing a significant difference between *c587-GAL4* and *cdc42* mutants in 29 ^o^C to 18 ^o^C but not in 18 ^o^C to 29 ^o^C (n = 50). (G) Quantification of the number of spectrosomes in 18 ^o^C to 29 ^o^C temperature shift flies in *c587-GAL4*, *dWnt4*, *RhoA*, *Rac1* and *cdc42* depleted escort cells showing a significant difference between *c587-GAL4* and *dWnt4* RNAi mutants, but not between *c587-GAL4* and *RhoA*, *Rac1* and *cdc42* mutants (n = 50). (H) Quantification of the number of spectrosomes in 29 ^o^C to 18 ^o^C temperature shift flies, in *c587-GAL4*, *dWnt4*, *RhoA*, *Rac1* and *cdc42* depleted escort cells showing a significant difference between *c587-GAL4* and *dWnt4*, *RhoA*, *Rac1* and *cdc42* depleted escort cells (n = 50). Scale bar for all images is 20μm.
(TIF)

S10 Fig Downstream Wnt non-canonical components regulate intermingling of ICs. (A-C) Larval gonads of *tj-GAL4* (control) and mutants expressing dominant negative *Rho* and *cdc42* in the ICs stained with Tj (red), 1B1 (blue) and Vasa (green) showing loss of intermingling of ICs in the mutants. (D-H) Larval gonads of control, *dsh*^*1*^, *cdc42*^*5*^, *cdc42 (cdc42*^*2*^*/cdc42*^*5*^*)* mutants and *Rac1*, *Rac2*, *Mtl* heterozygotes stained with Tj (red), 1B1 (blue) and Vasa (green) showing loss of intermingling of ICs in the mutants and *Rac* heterozygotes. Scale bar for all images is 20μm.
(TIF)

S11 Fig Downstream Wnt non-canonical components, *RhoA*, *Rac1*, *Mtl* and *cdc42* regulate IC cell division and *RhoA* regulates cell survival. (A-H) Larval gonads of control, *dWnt4* heterozygous, *dWnt4* mutant, *tj-GAL4* (control), *RhoA*, *Rac1*, *Mtl* and *cdc42* depleted ICs stained with Tj (red), PH3 (green) and 1B1 (blue) showing a significant difference in the division rate of the ICs between control and *dWnt4* mutants and, *tj-GAL4* and *RhoA*, *Rac1*, *Mtl* and *cdc42* mutants (n = 10). (I-O) Larval gonads of *dWnt4* heterozygous, *dWnt4* mutants, *tj-GAL4* (control), *RhoA*, *Rac1*, *Mtl* and *cdc42* depleted ICs stained with Tj (red), Caspase3 (green) and 1B1 (blue) showing Caspase3 positive staining in gonads with *RhoA* depleted ICs (white arrows) (n = 5). Scale bar for all images is 20μm.
(TIF)

S12 Fig PCP regulators and Wnt canonical pathway component do not regulate the intermingling of ICs. (A-F) Larval gonads of *tj-GAL4* (control), *vang*, *ds*, *fz*, *ft* and *arr* depleted ICs stained with Tj (red), 1B1 (blue) and Vasa (green) showing no intermingling defects in *vang*, *ds*, *fz*, *ft* and *arr* depleted ICs (n = 25). Scale bar for all images is 20μm.
(TIF)

S13 Fig *dWnt4* does not regulate the protein levels of RhoA, Rac1 and cdc42 in the ICs. (A-F1) Larval gonad of *dWnt4* heterozygous and *dWnt4* mutants that carry GFP tagged to RhoA, Rac1 or cdc42 stained for Tj (red), GFP (green) and Vasa (blue) showing similar expression of RhoA, Rac1 or cdc42 in the ICs of heterozygotes and mutants. GFP channel is shown in A1, B1, C1, D1, E1 and F1. (G) Quantification (n = 3) of GFP in the ICs showing that the protein levels of RhoA, Rac1 and cdc42 are not altered between *dWnt4* heterozygotes and *dWnt4* mutants. (H-H1) Larval gonad of *dWnt4* heterozygous lacking RhoA/Rac GTPase reporter, imaged under the same confocal settings (Fig 6A–6B1) showing no GFP. GFP channel is shown in H1. (I-I1) Larval gonad of *dWnt4* heterozygous lacking cdc42 GTPase reporter, imaged under the same confocal settings (Fig 6C–6D1) showing no GFP. GFP channel is shown in I1 (J) Quantification (n = 3) of GFP in the terminal filament showing that cdc42 activity is not altered in *dWnt4* mutants. Scale bar for all images is 20μm.
(TIF)
