# Supplementary figures and images for: A switch in the mode of Wnt signaling orchestrates the formation of germline stem cell differentiation niche in Drosophila
Source: PLoS Genet. 2018 Jan 25;14(1):e1007154. doi: 10.1371/journal.pgen.1007154 (PMC5811049; doi:10.1371/journal.pgen.1007154)

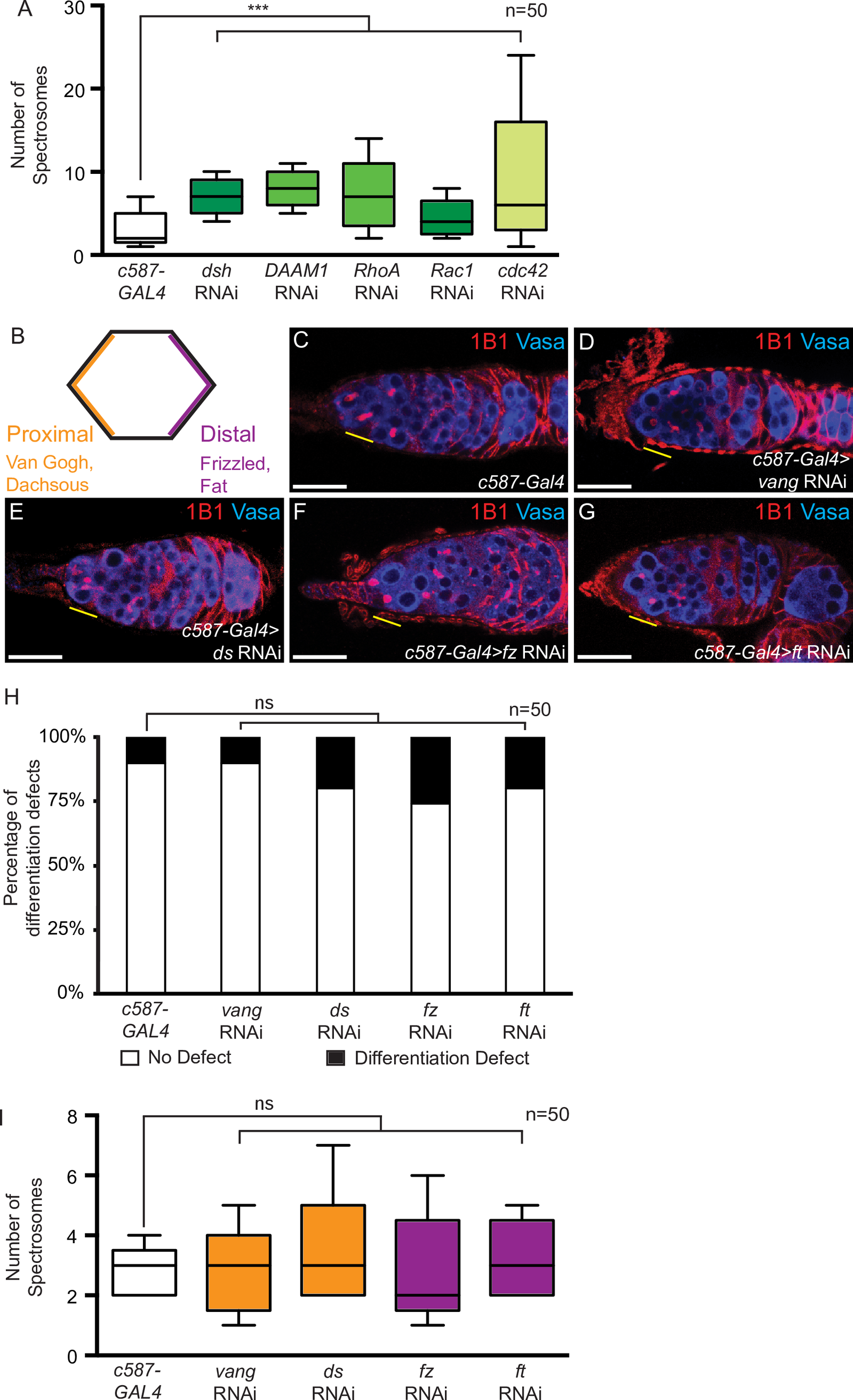

Supplement: S1 Fig — (A) Quantification of spectrosomes in c587-GAL4, dsh, DAAM1, RhoA, Rac1 and cdc42 depleted escort cells showing a significant difference in mutants (n = 50). (B) A schematic showing proteins that regulate the PCP of a cell. (C-G) Germaria of c587-GAL4 (control), vang, ds, fz and ft depleted escort cells stained with 1B1 (red) and Vasa (blue) showing no accumulation of undifferentiated cells in vang, ds, fz and ft depleted escort cells (yellow line). (H) Percentage of the germaria with >3 spectrosomes in c587-GAL4, vang, ds, fz and ft depleted escort cells showing no difference in vang, ds, fz and ft depleted escort cells (n = 50). (I) Quantification of spectrosomes in c587-GAL4, vang, ds, fz and ft depleted escort cells showing no significant difference (n = 50). Scale bar for all images is 20μm. (TIF) [file pgen.1007154.s001.tif]

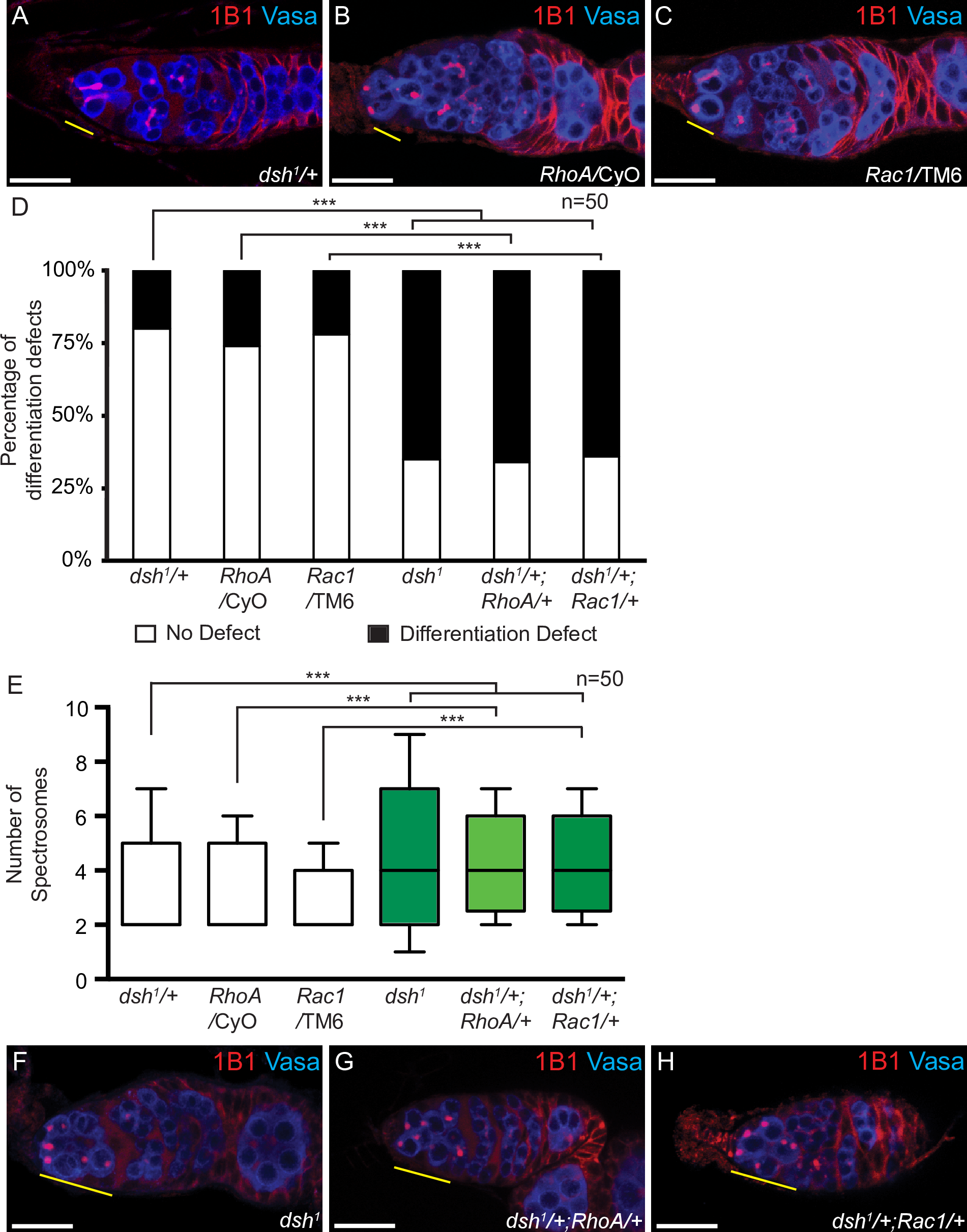

Supplement: S2 Fig — (A-C) dsh1/+, RhoA/CyO, and Rac1/TM6 stained with 1B1 (red) and Vasa (blue) showing no accumulation of undifferentiated cells (yellow line). (D) Percentage of the germaria with >3 spectrosomes in dsh1/+, RhoA/CyO, Rac1/TM6, dsh1 mutants, dsh1/RhoA trans-heterozygote and dsh1/Rac1 trans-heterozygote showing a significant difference in differentiation defects in the trans-heterozygotes (n = 50). (E) Quantification of the number of spectrosomes in dsh1/+, RhoA/CyO, Rac1/TM6, dsh1 mutants, dsh1/RhoA trans-heterozygote and dsh1/Rac1 trans-heterozygote showing a significant difference in the number of spectrosomes in the trans-heterozygotes (n = 50). (F-H) dsh1 mutants, dsh1/RhoA trans-heterozygote and dsh1/Rac1 trans-heterozygote stained with 1B1 (red) and Vasa (blue) showing an accumulation of >3 undifferentiated cells in the trans-heterozygotes (yellow line). Scale bar for all images is 20μm. (TIF) [file pgen.1007154.s002.tif]

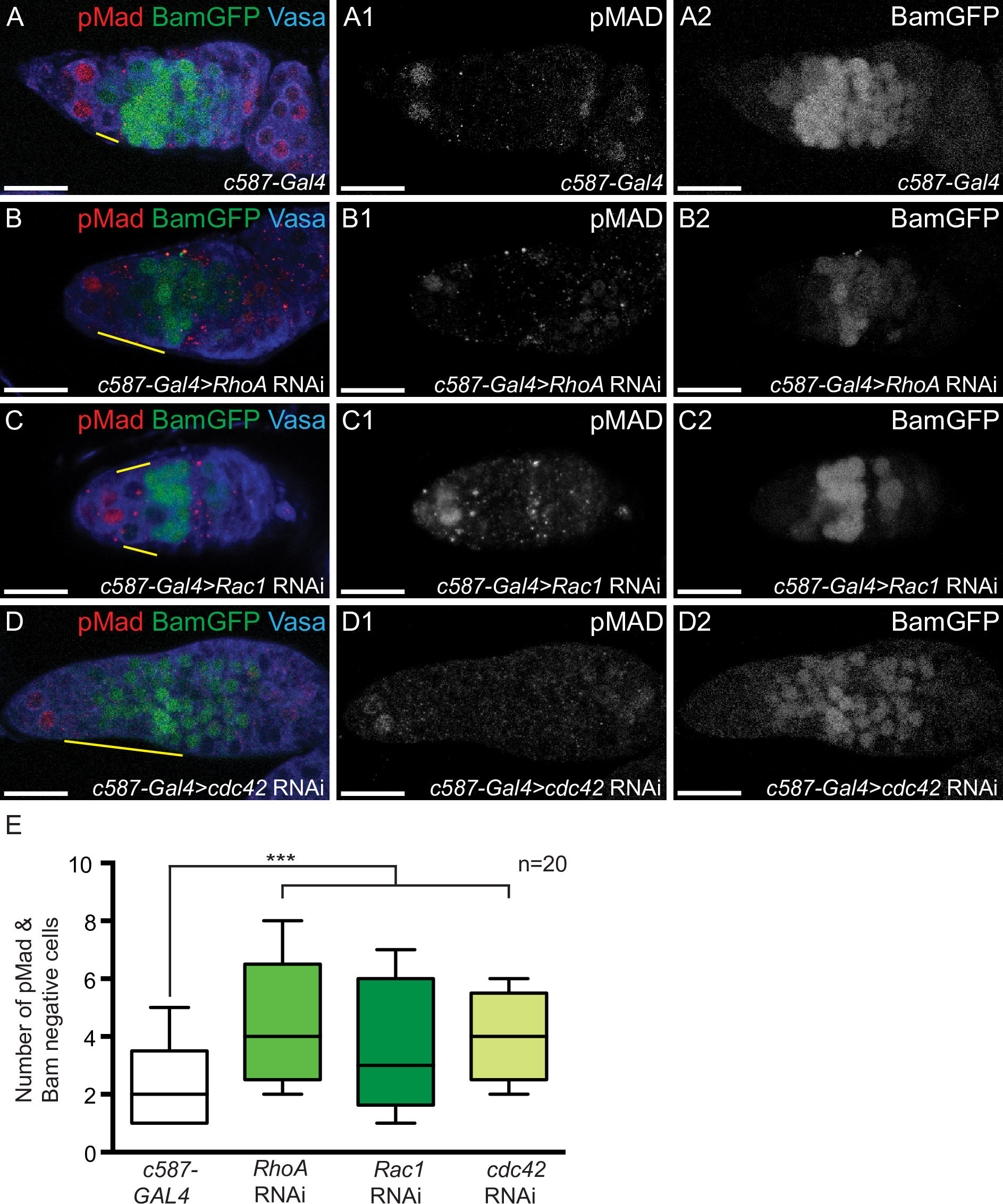

Supplement: S3 Fig — (A-D2) Germaria of c587-GAL4 (control), RhoA, Rac1 and cdc42 depleted escort cells stained with pMAD (red), GFP (green) and Vasa (blue) showing an accumulation of >3 pMAD and Bam negative cells in RhoA, Rac1 and cdc42 mutants (yellow line). pMAD channel is shown in A1, B1, C1 and D1; GFP channel is shown in A2, B2, C2 and D2. (E) Quantification of number of pMAD and Bam negative cells in c587-GAL4, RhoA, Rac1 and cdc42 depleted escort cells showing a significant increase in pMAD and Bam negative cells in RhoA, Rac1 and cdc42 mutants (n = 20). Scale bar for all images is 20μm. (TIF) [file pgen.1007154.s003.tif]

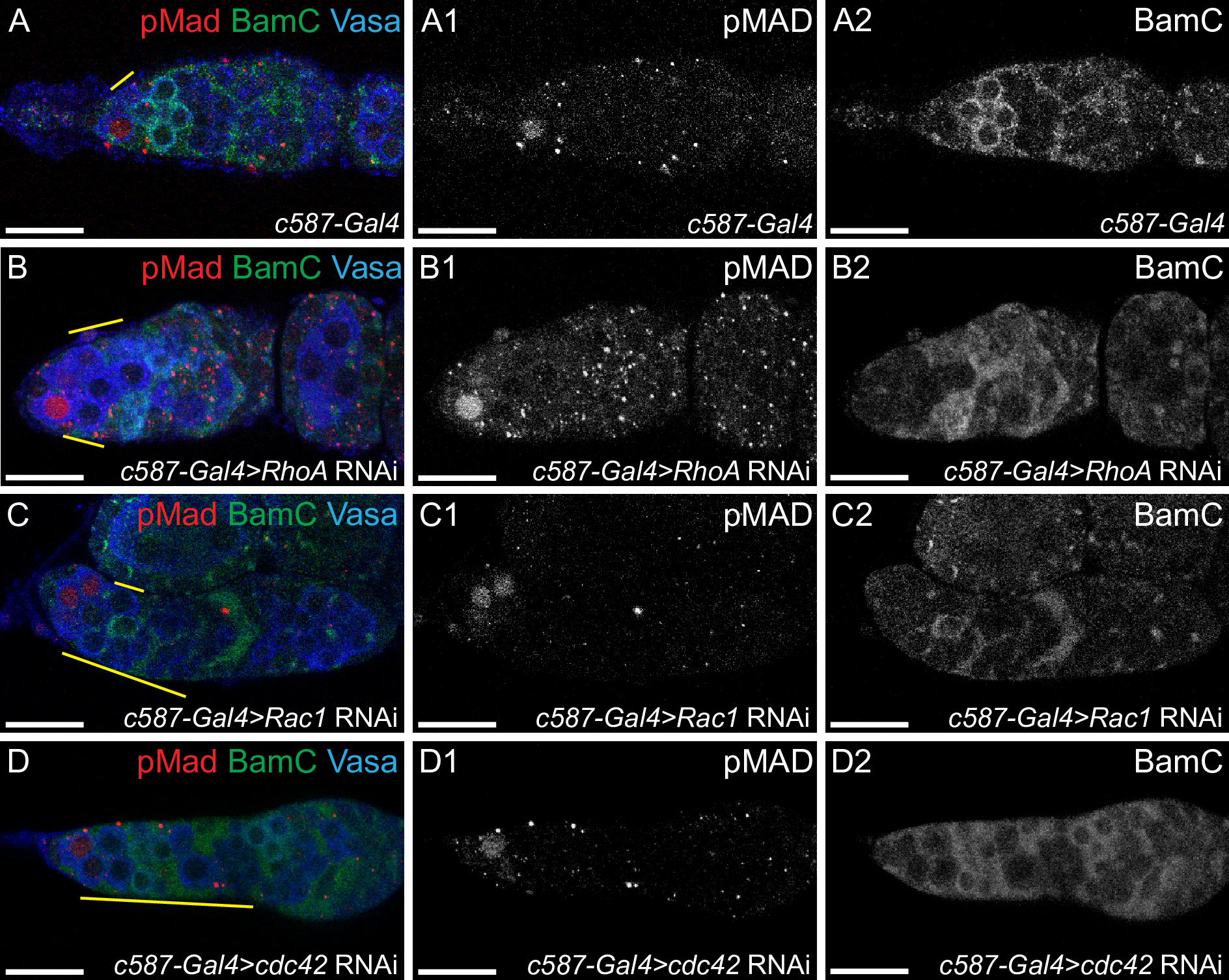

Supplement: S4 Fig — (A-D2) Germaria of c587-GAL4 (control), RhoA, Rac1 and cdc42 depleted escort cells stained with pMAD (red), BamC (green) and Vasa (blue) showing an accumulation of >3 pMAD and BamC negative cells in RhoA, Rac1 and cdc42 mutants (yellow line). pMAD channel is shown in A1, B1, C1 and D1; BamC channel is shown in A2, B2, C2 and D2. Scale bar for all images is 20μm. (TIF) [file pgen.1007154.s004.tif]

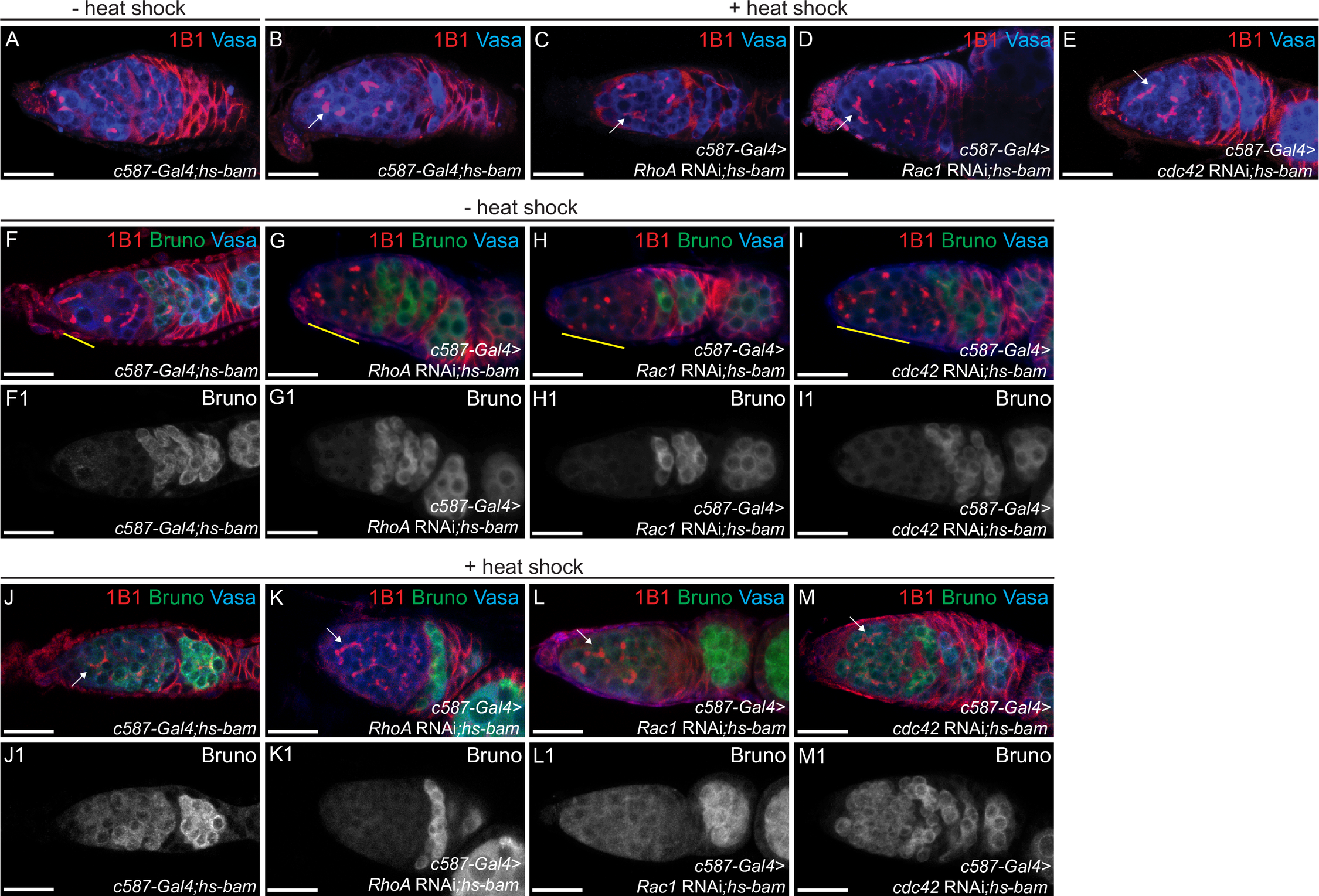

Supplement: S5 Fig — (A) c587-GAL4 (control) carrying a hs-bam transgene stained with 1B1 (red) and Vasa (blue) without heat-shock. (B-E) c587-GAL4 (control), RhoA, Rac1 and cdc42 depleted escort cell mutants carrying a hs-bam transgene stained with 1B1 (red) and Vasa (blue) showing differentiating cysts marked by fusomes (red) (white arrow) and a lack of undifferentiated cells post heat-shock. (F-I1) c587-GAL4 (control), RhoA, Rac1 and cdc42 depleted escort cell mutants carrying a hs-bam transgene, without heat-shock stained with 1B1 (red), Bruno (green) and Vasa (blue) showing accumulation of undifferentiated cells (yellow line) marked by the presence of spectrosomes (red) and low Bruno expression in the undifferentiated cells and early cysts, while high Bruno expression in 16-cell cyst and onwards. Bruno channel is shown in F1, G1, H1 and I1. (J-M1) c587-GAL4 (control), RhoA, Rac1 and cdc42 depleted escort cell mutants carrying a hs-bam transgene stained with 1B1 (red), Bruno (green) and Vasa (blue), post heat-shock showing Bruno expression in the differentiating cysts (white arrow) marked by fusomes (red) and a lack of undifferentiated cells post heat-shock. Cysts in post heat-shock Rho mutants showed weak Bruno staining. Bruno channel is shown in J1, K1, L1, and M1. Scale bar for all images is 20μm. (TIF) [file pgen.1007154.s005.tif]

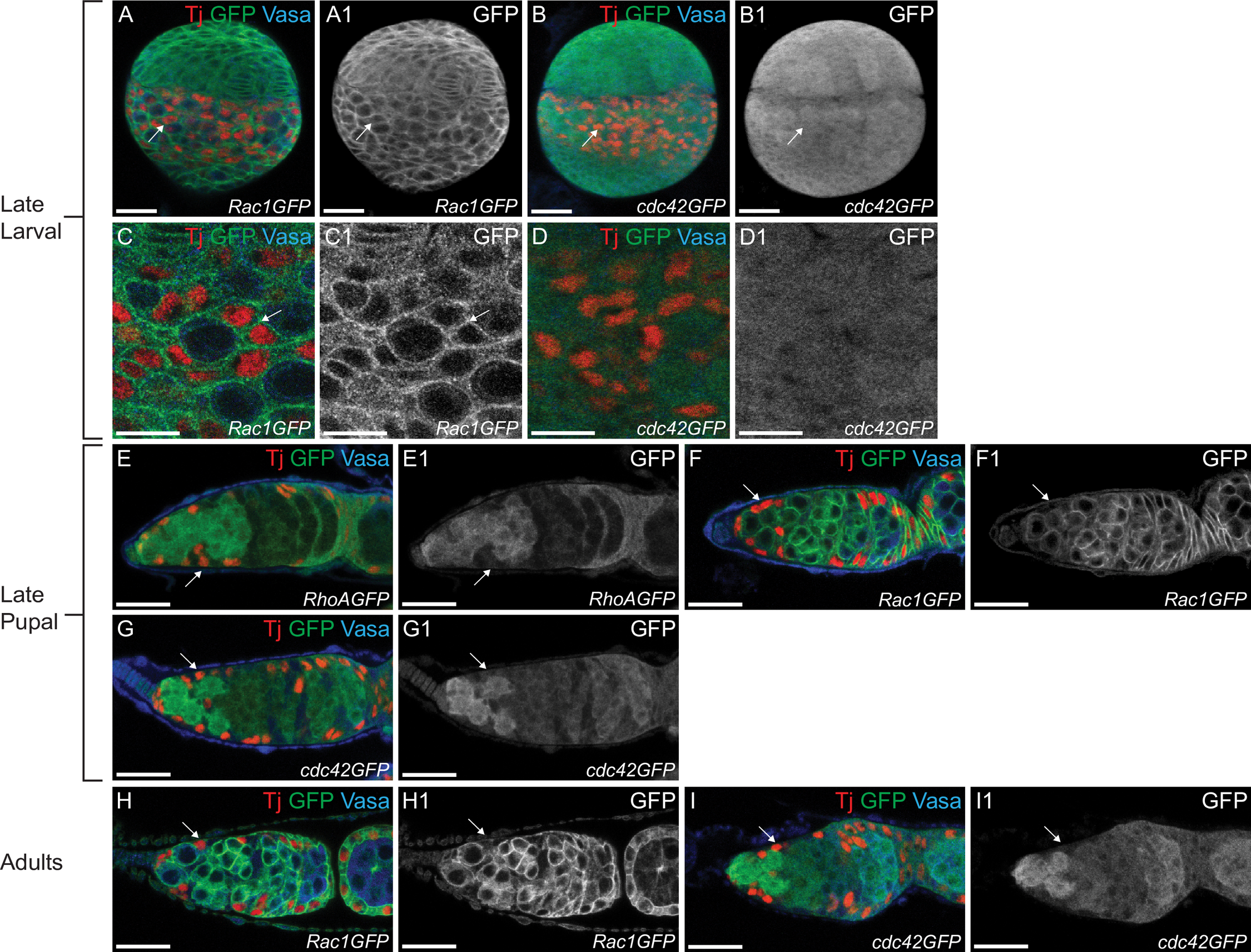

Supplement: S6 Fig — (A-D1) Late larval gonad of transgenic flies with GFP tagged to Rac1 and cdc42, respectively stained for Tj (red), Vasa (blue) and GFP (green) showing high expression of Rac1 and cdc42 in the ICs (white arrow). 63x is shown in C-C1 and D-D1. GFP channel is shown in A1, B1, C1 and D1. (E-G1) Late pupal germaria of transgenic flies with GFP tagged to RhoA, Rac1 and cdc42, respectively stained for Tj (red), GFP (green) and Vasa (blue) showing low expression of RhoA, Rac1, and cdc42 in pupal escort cells (white arrow). GFP channel is shown in E1, F1 and G1. (H-I1) Adult germaria of transgenic flies with GFP tagged to Rac1 and cdc42, respectively stained for Tj (red), GFP (green) and Vasa (blue) showing low expression of Rac1 and cdc42 in the adult escort cells (white arrow). GFP channel is shown in H1 and I1. Scale bar for C1 and D1 is 10μm. Scale bar for all other images is 20μm. (TIF) [file pgen.1007154.s006.tif]

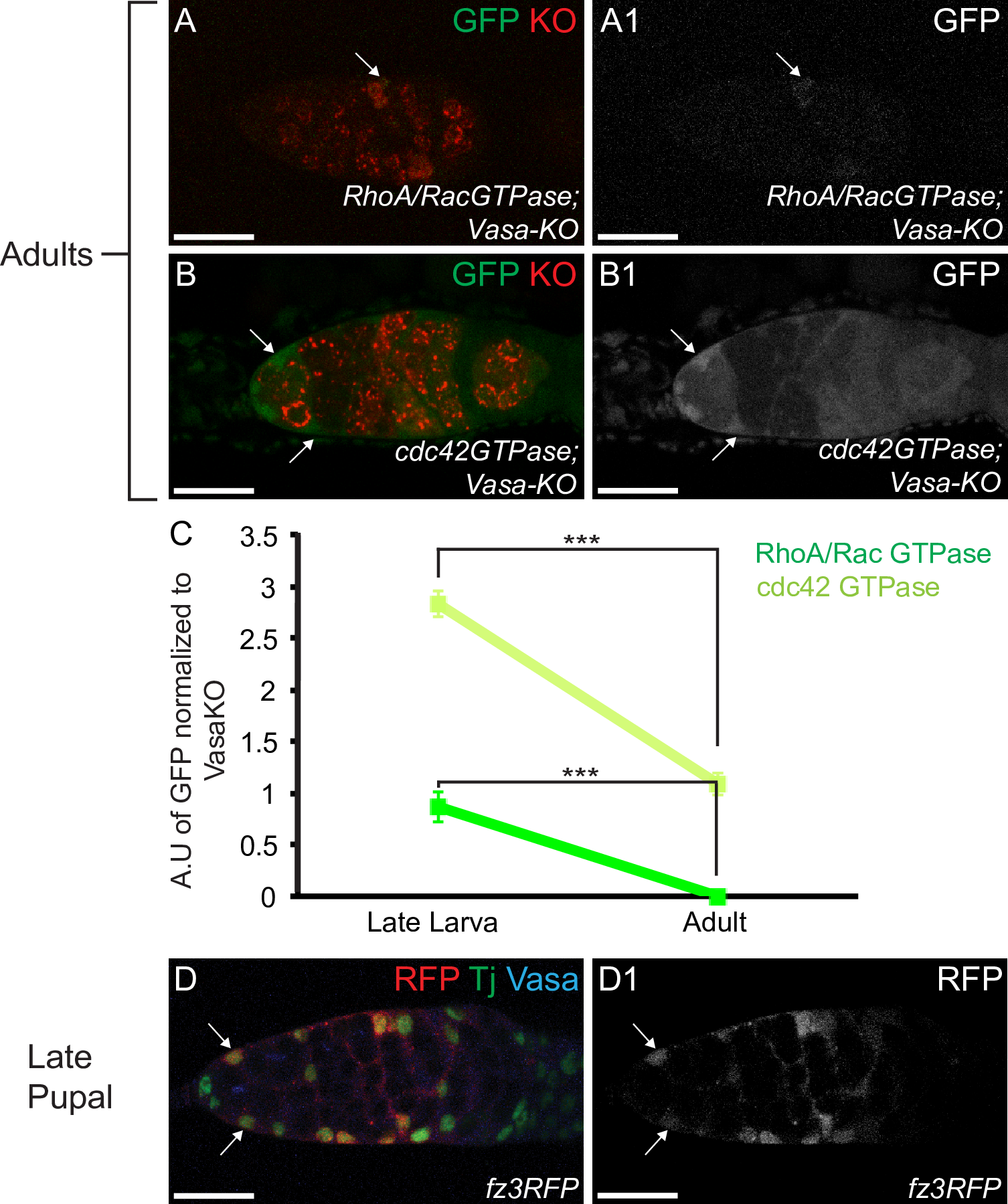

Supplement: S7 Fig — (A-B1) Adult germaria of transgenic flies that report active form of RhoA/Rac and cdc42. While RhoA/Rac is not expressed in the escort cells, it was expressed in the follicle cells (white arrow). cdc42 is active in the adult escort cells. Germline is marked by Vasa-KO. GFP channels are shown in A1 and B1. (C) Quantification (n = 3) of GFP in the ICs and adult escort cells showing that while active RhoA/Rac is not expressed in the adult escort cells, active form of cdc42 is highly expressed in the ICs in comparison to the adult escort cells. (D-D1) Late pupal germarium of fly carrying the Wnt canonical reporter, Frizzled3 (Fz3) stained for RFP (red), Tj (green) and 1B1 (blue) showing high expression of Fz3 in the pupal escort cells (white arrow). RFP channel is shown in D1. Scale bar for all images is 20μm. (TIF) [file pgen.1007154.s007.tif]

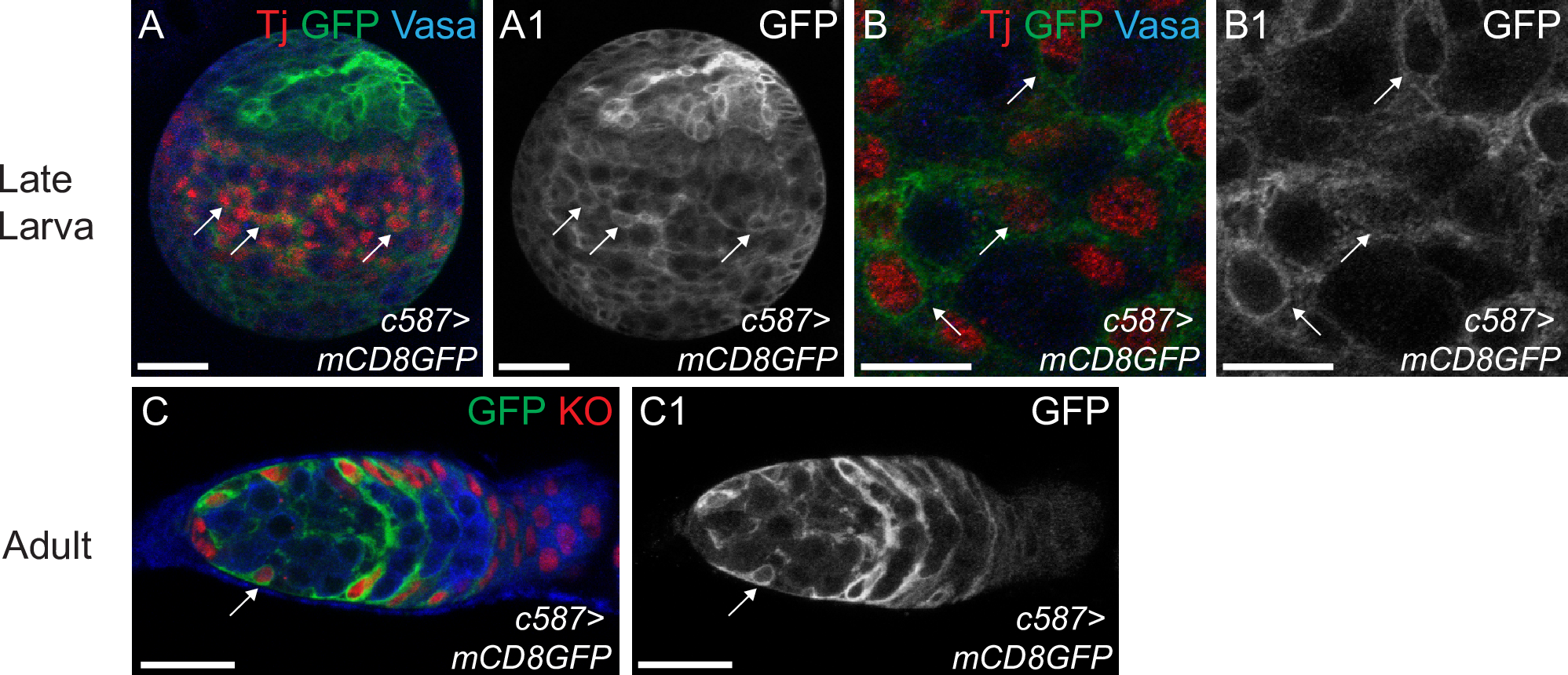

Supplement: S8 Fig — (A-B1) Late larval gonad of flies with mCD8GFP under the control of c587 stained for Tj (red), GFP (green) and Vasa (blue) showing GFP expression in the ICs (white arrows). 63x is shown in B-B1. GFP channel is shown in A1 and B1. (C-C1) Adult germaria of flies with mCD8GFP under the control of c587 stained for Tj (red), GFP (green) and Vasa (blue) showing GFP expression in the escort cells (white arrow). GFP channel is shown in C1. Scale bar for B and B1 is 10μm. Scale bar for all other images is 20μm. (TIF) [file pgen.1007154.s008.tif]

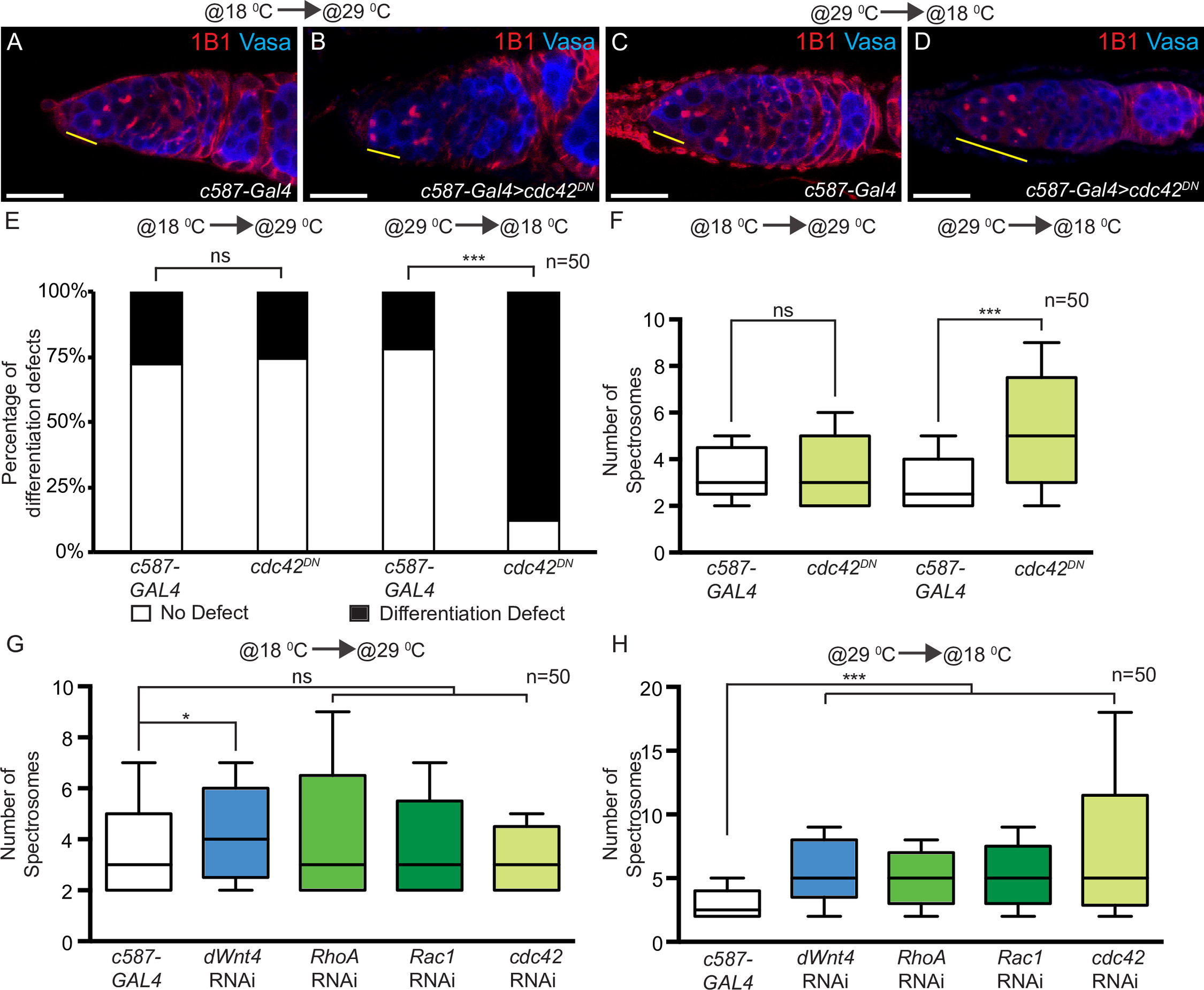

Supplement: S9 Fig — (A-B) Germaria of c587-GAL4 (control) and 18 oC to 29 oC temperature shift cdc42 depleted escort cells using cdc42DN stained with 1B1 (red) and Vasa (blue) showing no differentiation defects in cdc42 mutants (yellow line). (C-D) Germaria of c587-GAL4 (control) and 29 oC to 18 oC temperature shift cdc42 depleted escort cells using cdc42DN stained with 1B1 (red) and Vasa (blue) showing an accumulation of >3 undifferentiated cells in cdc42 mutants (yellow line). (E) Percentage of the germaria with >3 spectrosomes in 18 oC to 29 oC and 29 oC to 18 oC temperature shift flies, in c587-GAL4 and cdc42DN mutants showing a significant difference between c587-GAL4 and cdc42DN expressed escort cells in 29 oC to 18 oC but not in 18 oC to 29 oC (n = 50). (F) Quantification of the number of spectrosomes in 18 oC to 29 oC and 29 oC to 18 oC temperature shift flies in c587-GAL4, cdc42DN expressed escort cells showing a significant difference between c587-GAL4 and cdc42 mutants in 29 oC to 18 oC but not in 18 oC to 29 oC (n = 50). (G) Quantification of the number of spectrosomes in 18 oC to 29 oC temperature shift flies in c587-GAL4, dWnt4, RhoA, Rac1 and cdc42 depleted escort cells showing a significant difference between c587-GAL4 and dWnt4 RNAi mutants, but not between c587-GAL4 and RhoA, Rac1 and cdc42 mutants (n = 50). (H) Quantification of the number of spectrosomes in 29 oC to 18 oC temperature shift flies, in c587-GAL4, dWnt4, RhoA, Rac1 and cdc42 depleted escort cells showing a significant difference between c587-GAL4 and dWnt4, RhoA, Rac1 and cdc42 depleted escort cells (n = 50). Scale bar for all images is 20μm. (TIF) [file pgen.1007154.s009.tif]

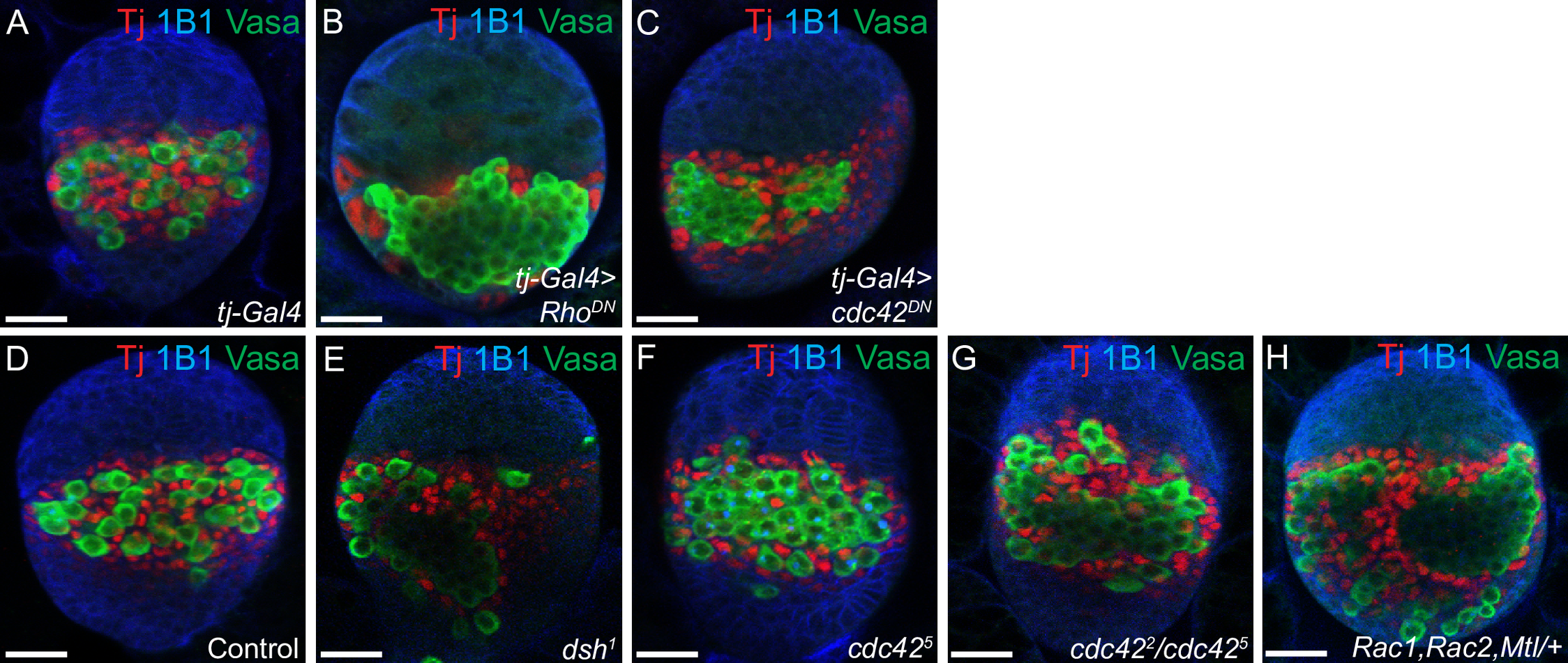

Supplement: S10 Fig — (A-C) Larval gonads of tj-GAL4 (control) and mutants expressing dominant negative Rho and cdc42 in the ICs stained with Tj (red), 1B1 (blue) and Vasa (green) showing loss of intermingling of ICs in the mutants. (D-H) Larval gonads of control, dsh1, cdc425, cdc42 (cdc422/cdc425) mutants and Rac1, Rac2, Mtl heterozygotes stained with Tj (red), 1B1 (blue) and Vasa (green) showing loss of intermingling of ICs in the mutants and Rac heterozygotes. Scale bar for all images is 20μm. (TIF) [file pgen.1007154.s010.tif]

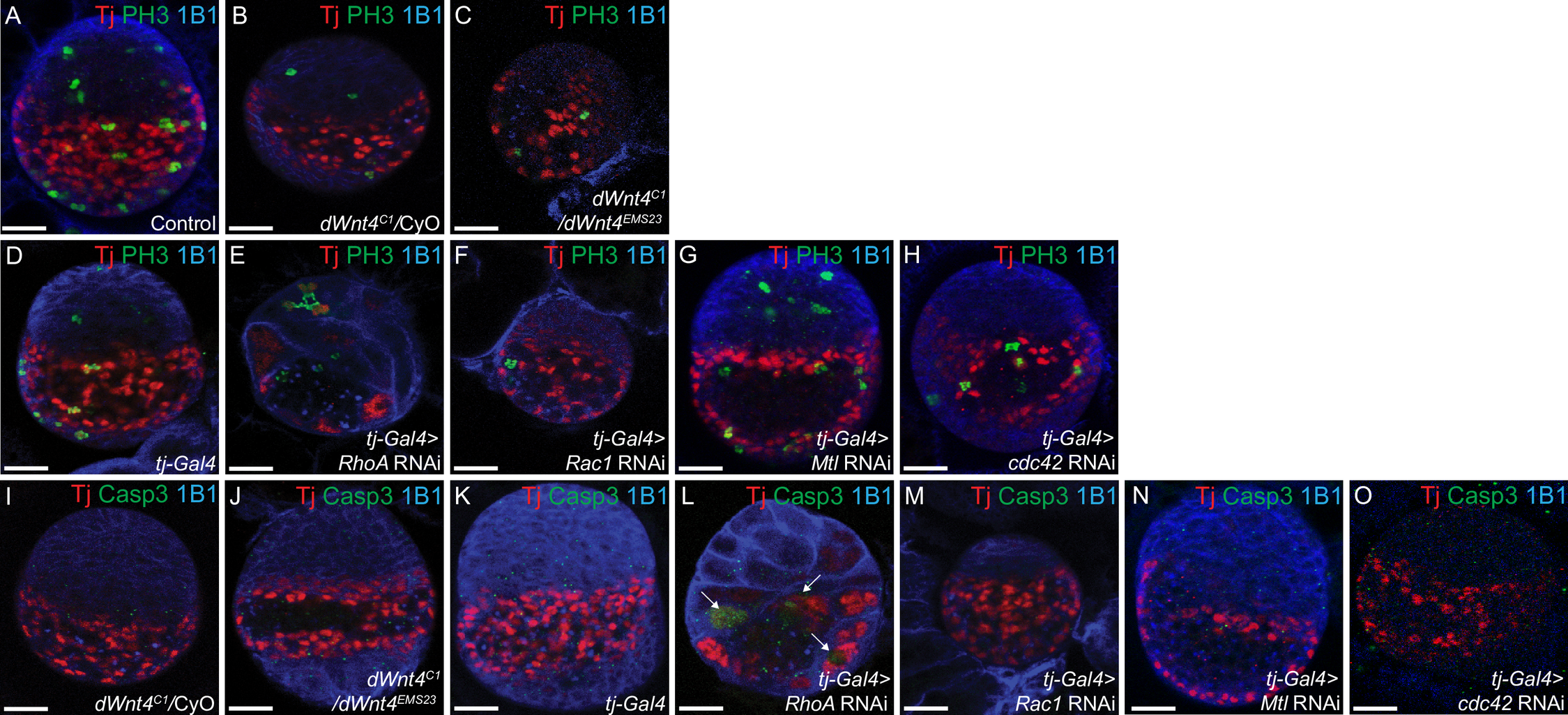

Supplement: S11 Fig — (A-H) Larval gonads of control, dWnt4 heterozygous, dWnt4 mutant, tj-GAL4 (control), RhoA, Rac1, Mtl and cdc42 depleted ICs stained with Tj (red), PH3 (green) and 1B1 (blue) showing a significant difference in the division rate of the ICs between control and dWnt4 mutants and, tj-GAL4 and RhoA, Rac1, Mtl and cdc42 mutants (n = 10). (I-O) Larval gonads of dWnt4 heterozygous, dWnt4 mutants, tj-GAL4 (control), RhoA, Rac1, Mtl and cdc42 depleted ICs stained with Tj (red), Caspase3 (green) and 1B1 (blue) showing Caspase3 positive staining in gonads with RhoA depleted ICs (white arrows) (n = 5). Scale bar for all images is 20μm. (TIF) [file pgen.1007154.s011.tif]

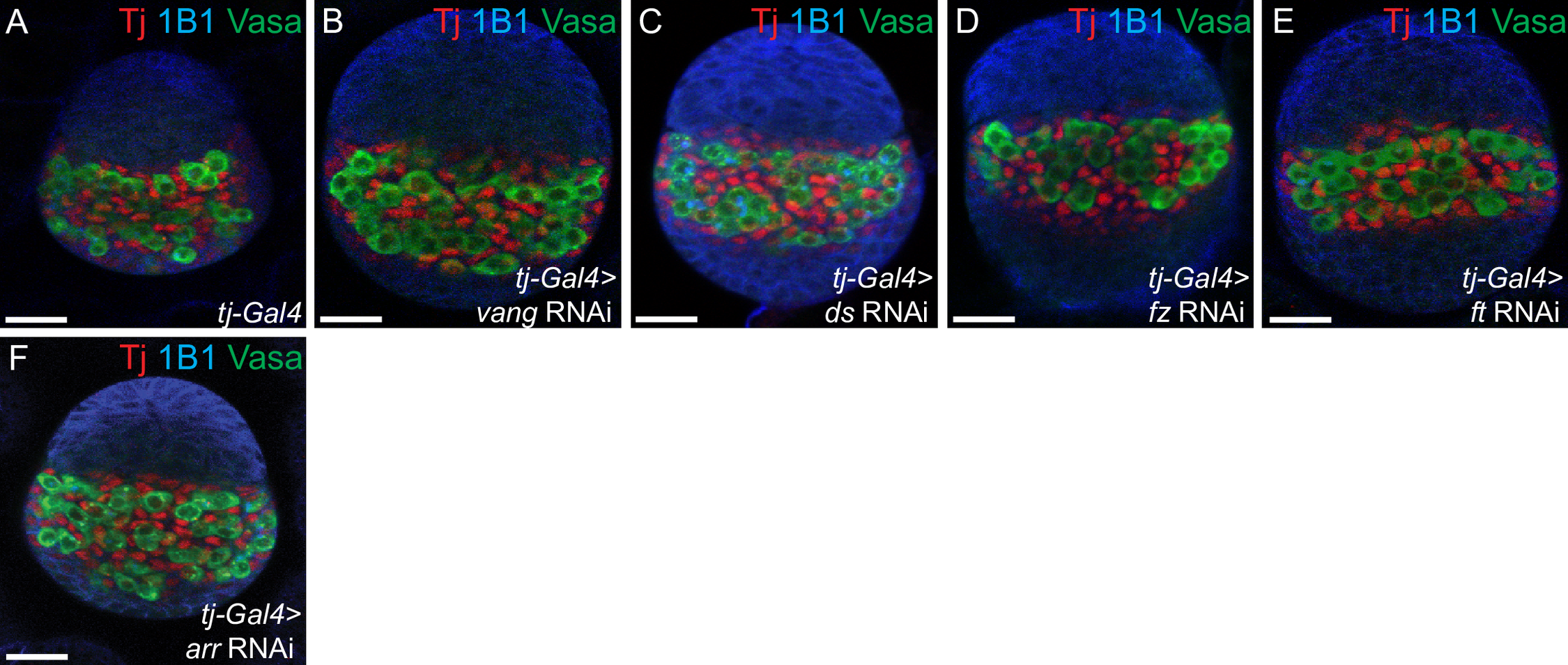

Supplement: S12 Fig — (A-F) Larval gonads of tj-GAL4 (control), vang, ds, fz, ft and arr depleted ICs stained with Tj (red), 1B1 (blue) and Vasa (green) showing no intermingling defects in vang, ds, fz, ft and arr depleted ICs (n = 25). Scale bar for all images is 20μm. (TIF) [file pgen.1007154.s012.tif]

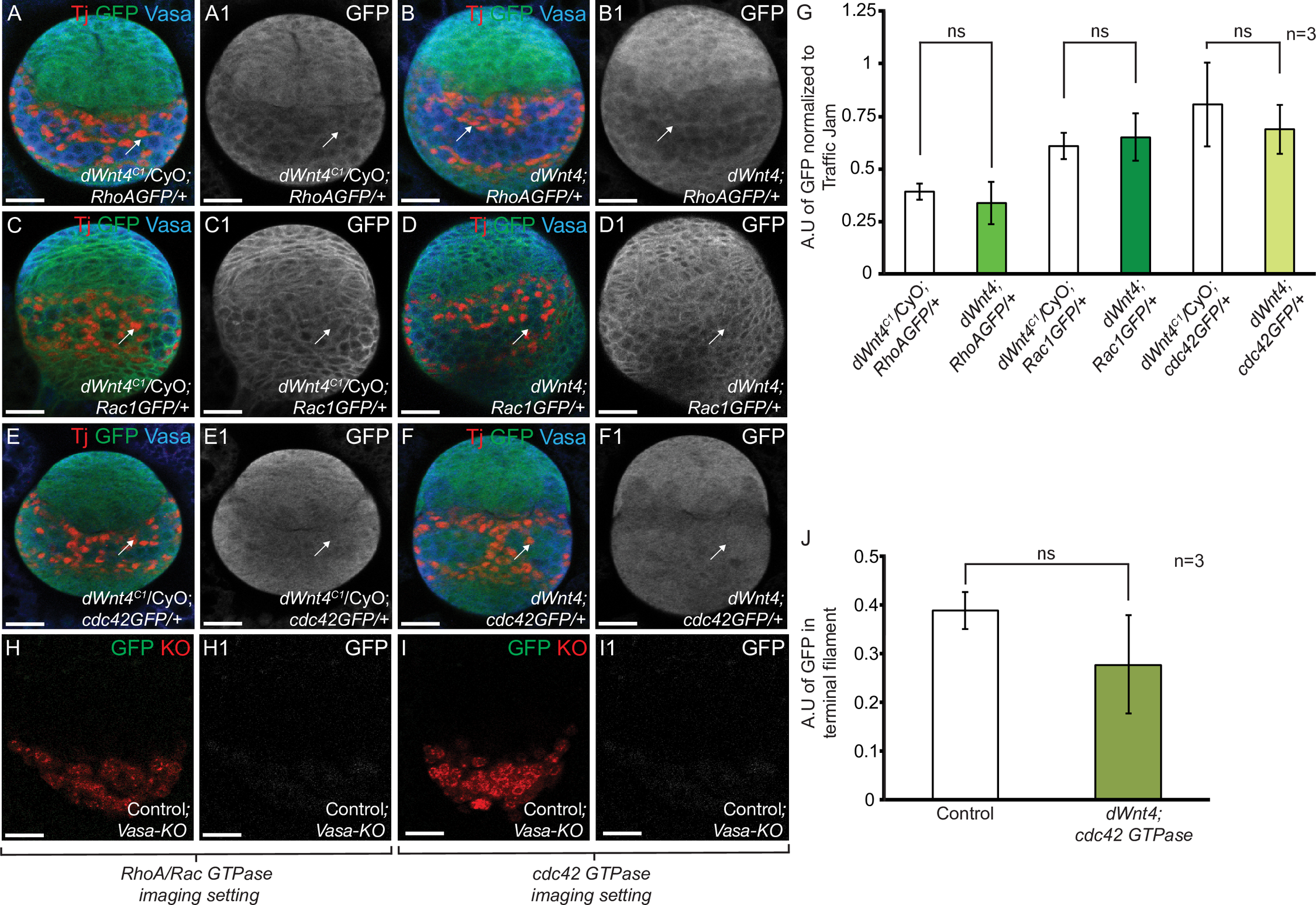

Supplement: S13 Fig — (A-F1) Larval gonad of dWnt4 heterozygous and dWnt4 mutants that carry GFP tagged to RhoA, Rac1 or cdc42 stained for Tj (red), GFP (green) and Vasa (blue) showing similar expression of RhoA, Rac1 or cdc42 in the ICs of heterozygotes and mutants. GFP channel is shown in A1, B1, C1, D1, E1 and F1. (G) Quantification (n = 3) of GFP in the ICs showing that the protein levels of RhoA, Rac1 and cdc42 are not altered between dWnt4 heterozygotes and dWnt4 mutants. (H-H1) Larval gonad of dWnt4 heterozygous lacking RhoA/Rac GTPase reporter, imaged under the same confocal settings (Fig 6A–6B1) showing no GFP. GFP channel is shown in H1. (I-I1) Larval gonad of dWnt4 heterozygous lacking cdc42 GTPase reporter, imaged under the same confocal settings (Fig 6C–6D1) showing no GFP. GFP channel is shown in I1 (J) Quantification (n = 3) of GFP in the terminal filament showing that cdc42 activity is not altered in dWnt4 mutants. Scale bar for all images is 20μm. (TIF) [file pgen.1007154.s013.tif]
